# Supplementary material for: Behaviour change physiotherapy intervention to increase physical activity following hip and knee replacement (PEP-TALK): study protocol for a pragmatic randomised controlled trial
Source: BMJ Open. 2020 Jul 19;10(7):e035014. doi: 10.1136/bmjopen-2019-035014 (PMC7371148; doi:10.1136/bmjopen-2019-035014)
Supplement: Supplementary data [file bmjopen-2019-035014supp001.pdf]

UNIVERSITY OF OXFORD

CONSENT FORM (PEP-TALK STUDY)

PEP-TALK

Name of Local Principal Investigator:________________________________

Screening Number: ☐☐ - ☐☐☐☐

LOCAL TRUST LOGO

If you agree, please initial

1. I confirm that I have read and understood the Information Leaflet dated 10 October 2019 version 4.0. I have had the opportunity to consider the information, ask questions and have had these answered satisfactorily.	
2. I understand that my participation is voluntary and that I am free to withdraw at any time without giving any reason, and without my medical care or legal rights being affected.	
3. I understand that relevant sections of my medical notes and data collected during the study may be looked at by individuals from the University of Oxford, from regulatory authorities [and from the NHS Trust(s)], where it is relevant to me taking part in this research. I give permission for these individuals to have access to my records.	
4. I consent to the research team holding my contact details so that they can contact me about the study. I understand these details will be held securely and destroyed at the end of the study.	
5. I am aware that treatment sessions may be observed for quality assurance purposes.	
6. I agree to my General Practitioner (GP) being informed of my participation in the study and questionnaire results.	
7. I agree to be contacted for the purposes of follow up by the central PEP-TALK team who are based in Oxford.	
8. I agree to take part in the PEP-TALK study.	

Name of Participant **Date** **Signature**

________________ ________________ ________________

Name of Person Taking Consent **Date** **Signature**

________________ ________________ ________________

STUDY RESULTS – THIS SECTION IS OPTIONAL TO COMPLETE

I agree to be contacted about ethically approved research studies for which I may be suitable. I understand that agreeing to be contacted does not oblige me to participate in any further studies.

YES ☐ NO ☐
