# Supplementary material for: Behaviour change physiotherapy intervention to increase physical activity following hip and knee replacement (PEP-TALK): study protocol for a pragmatic randomised controlled trial
Source: BMJ Open. 2020 Jul 19;10(7):e035014. doi: 10.1136/bmjopen-2019-035014 (PMC7371148; doi:10.1136/bmjopen-2019-035014)
Supplement: Author's manuscript [file bmjopen-2019-035014.draft_revisions.pdf]

BMJ Open is committed to open peer review. As part of this commitment we make the peer review history of every article we publish publicly available.

When an article is published we post the peer reviewers' comments and the authors' responses online. We also post the versions of the paper that were used during peer review. These are the versions that the peer review comments apply to.

The versions of the paper that follow are the versions that were submitted during the peer review process. They are not the versions of record or the final published versions. They should not be cited or distributed as the published version of this manuscript.

BMJ Open is an open access journal and the full, final, typeset and author-corrected version of record of the manuscript is available on our site with no access controls, subscription charges or pay-per-view fees (http://bmjopen.bmj.com).

If you have any questions on BMJ Open's open peer review process please email info.bmjopen@bmj.com

BMJ Open

Behaviour change physiotherapy intervention to increase physical activity following hip and knee replacement (PEP-TALK): study protocol for a pragmatic randomised controlled trial

Journal:	*BMJ Open*
Manuscript ID	bmjopen-2019-035014
Article Type:	Protocol
Date Submitted by the Author:	15-Oct-2019

Complete List of Authors:	Smith, Toby O.; University of Oxford , Nuffield Department of Orthopaedics, Rheumatology and Musculoskeletal Sciences; University of East Anglia, Faculty of Medicine and Health Sciences<br>Parsons, Scott; University of Oxford, Nuffield Department of Orthopaedics, Rheumatology and Musculoskeletal Disorders<br>Fordham, Beth; University of Oxford , NDORMS<br>Ooms, Alexander; University of Oxford, Nuffield Department of Orthopaedics, Rheumatology and Musculoskeletal Disorders<br>Dutton, Susan; University of Oxford, CSM<br>Hing, Caroline; University of London St George's Molecular and Clinical Sciences Research Institute<br>Barber, Vicki; University of Oxford Nuffield Department of Orthopaedics Rheumatology and Musculoskeletal Sciences, Oxford Clinical Trials Unit<br>Png, May ee; University of Oxford, NDORMS<br>Lamb, Sarah; University of Oxford, Ndorms; University of Exeter, College of Medicine and Health Sciences

Keywords:	Adult orthopaedics < ORTHOPAEDIC & TRAUMA SURGERY, Hip < ORTHOPAEDIC & TRAUMA SURGERY, Knee < ORTHOPAEDIC & TRAUMA SURGERY

SCHOLARONE™
Manuscripts

I, the Submitting Author has the right to grant and does grant on behalf of all authors of the Work (as defined in the below author licence), an exclusive licence and/or a non-exclusive licence for contributions from authors who are: i) UK Crown employees; ii) where BMJ has agreed a CC-BY licence shall apply, and/or iii) in accordance with the terms applicable for US Federal Government officers or employees acting as part of their official duties; on a worldwide, perpetual, irrevocable, royalty-free basis to BMJ Publishing Group Ltd ("BMJ") its licensees and where the relevant Journal is co-owned by BMJ to the co-owners of the Journal, to publish the Work in this journal and any other BMJ products and to exploit all rights, as set out in our licence.

The Submitting Author accepts and understands that any supply made under these terms is made by BMJ to the Submitting Author unless you are acting as an employee on behalf of your employer or a postgraduate student of an affiliated institution which is paying any applicable article publishing charge ("APC") for Open Access articles. Where the Submitting Author wishes to make the Work available on an Open Access basis (and intends to pay the relevant APC), the terms of reuse of such Open Access shall be governed by a Creative Commons licence – details of these licences and which Creative Commons licence will apply to this Work are set out in our licence referred to above.

Other than as permitted in any relevant BMJ Author's Self Archiving Policies, I confirm this Work has not been accepted for publication elsewhere, is not being considered for publication elsewhere and does not duplicate material already published. I confirm all authors consent to publication of this Work and authorise the granting of this licence.

For peer review only

BMJ OPEN

TITLE PAGE

Title: Behaviour change physiotherapy intervention to increase physical activity following hip and knee replacement (PEP-TALK): study protocol for a pragmatic randomised controlled trial

Authors: Smith TO,[1,2] Parsons SR,[1] Fordham B,[1] Ooms AG,[1] Dutton SJ,[1] Hing CB,[3] Barber VS,[1] Png ME,[1] Lamb SE[1,4] on behalf of the PEP-TALK Trial Collaborators.

Affiliations

1. Nuffield Department of Rheumatology, Orthopaedics and Musculoskeletal Sciences, University of Oxford, Botnar Research Centre, Windmill Road, Oxford, UK
2. Faculty of Medicine and Health Sciences, University of East Anglia, Norwich, UK
3. Department of Trauma and Orthopaedics, St George's Hospital, St George's University Hospitals NHS Foundation Trust, Tooting, London, UK
4. College of Medicine and Health Sciences, University of Exeter, Exeter, UK

Corresponding Author: Dr Toby Smith, Nuffield Department of Rheumatology, Orthopaedics and Musculoskeletal Sciences, University of Oxford, Botnar Research Centre, Windmill Road, Oxford, UK. Email: toby.smith@ndorms.ox.ac.uk

Word Count: 4360

For peer review only

ABSTRACT

INTRODUCTION: Whilst total hip (THR) and knee replacements (TKR) successfully reduce pain associated with chronic joint pathology, this infrequently translates into increased physical activity. This is a challenge given that over 50% of individuals who undergo these operations are physically inactive and have medical comorbidities such as hypertension, heart disease, diabetes and depression. The impact of these diseases can be reduced with physical activity. This trial aims to investigate the effectiveness of a behaviour change physiotherapy intervention to increase physical activity compared to usual rehabilitation after THR or TKR.

METHODS AND ANALYSIS: The PEP-TALK trial is a multi-centre, open-labelled, pragmatic randomised controlled trial. 260 adults who are scheduled to undergo a primary unilateral THR or TKR and are moderately inactive or inactive, with comorbidities, will be recruited across eight sites in England. They will be randomised post-surgery, prior to hospital discharge, to either six, 30-minute weekly group-based exercise sessions(control), or the same six-weekly, group-based, exercise sessions each preceded by a 30-minute cognitive behaviour approach discussion group. Participants will be followed-up to 12 months by postal questionnaire. The primary outcome is the University of California Los Angeles (UCLA) Physical Activity Score at 12 months. Secondary outcomes include: physical function, disability, health-related quality of life, kinesiophobia, perceived pain, self-efficacy and health resource utilisation.

ETHICS AND DISSEMINATION: Research ethics committee (REC) approval was granted by the NRES Committee South Central (Oxford B - 18/SC/0423). Dissemination of results will be through peer-reviewed, scientific journals and conference presentations.

TRIAL ISRCTN REGISTRATION NUMBER: ISRCTN29770908

PROTOCOL VERSION: Version 4.0 – Date: 17th September 2019

STATUS: trial recruitment is ongoing and is expected to be completed by 1st April 2020.

Keywords: arthroplasty; osteoarthritis; rehabilitation; physical activity; exercise; cognitive behavioural

STRENGTHS AND LIMITATIONS OF THIS STUDY

- The effectiveness of a behaviour change physiotherapy intervention to increase physical activity compared to usual rehabilitation after THR or TKR will be demonstrated with a pragmatic clinical trial design.
- Functional, behavioural and psychological outcomes will provide evidence to determine the mechanisms by-which the intervention is or is not effective.
- A multi-centre recruitment approach will provide greater external validity across population characteristics in England.

For peer review only

BACKGROUND

Total Hip (THR) and Total Knee Replacement (TKR) are two highly successful orthopaedic procedures which reduce pain for people with osteoarthritis.[1,2] Over 206,000 THR and TKRs were performed in the United Kingdom (UK) in 2018.[1] Approximately 90% of patients are satisfied following THR and TKR,[2] with significant improvements in pain and physical function after three to 12 months.[2,3]

Historically, it has been assumed that people become more active following THR or TKR through the amelioration of joint pain.[4] However, the current literature suggests physical activity, at best, remains the same from pre- to post-operatively, and in some instances declines.[4,5]

People following THR and TKR have a number of challenges which make engaging in physical activity difficult, most notably psychosocial barriers and fear avoidance beliefs.[6] Such barriers include receiving insufficient and inconsistent information on being more physically active, fear of damaging joint replacements and causing pain, and not being able to goal-set or problem-solve physical activities within individual's lifestyles.[6]

Not being physically active after joint replacement can have a major negative impact on a person's health and a burden on the National Health Service (NHS). Medical co-morbidities are common in this population. These include hypertension (56%),[7] cardiovascular disease (20%),[8] diabetes (16%)[8] and multi-joint pain (57%).[7] Twenty-seven percent of people who undergo joint replacement have three to four comorbidities.[8] Medical comorbidities have a significant negative impact on both health-related quality of life (HRQoL) and result in a societal burden.[9,10] Participating in regular physical activity can decrease the risk of cardiovascular disease by 52%,[11] diabetes by 65%,[12] and some cancers by 40%.[13] It is associated with a reduction in all-cause mortality by 33% and cardiovascular mortality by 35%.[14]

Current rehabilitation following THR and TKR in the UK, as advocated by the British Orthopaedic Association, centres around regaining joint movement, strength and gait (walking pattern) re-education.[15] There is currently no evidence informing patients or healthcare professionals on how to increase physical activity following THR and TKR. Previous research has demonstrated that behaviour-change interventions can effectively increase physical activity across the lifespan.[16-18] However, following joint replacement, people have specific psychological needs and challenges which differ to the non-joint replacement population.[6] Therefore, a specific intervention tailored to this population's health beliefs, including fear avoidance regarding implant survival, dislocation and increased knowledge on the impact of physical inactivity on other comorbidities, is required. Accordingly, the purpose of this trial is to answer the research question "does a group exercise and behaviour-change intervention targeted to increase physical activity participation increase HRQoL and clinical outcomes following a THR or TKR?"

METHODS AND DESIGN

Trial Design

This is a two-arm, pragmatic, parallel, randomised controlled superiority trial (RCT) to assess the effectiveness of a group exercise and behaviour-change intervention aimed to increase physical

For peer review only

activity in people following THR or TKR. The study flow chart is presented as **Figure 1**. **Table 1** presents a summary of trial objectives, outcome measures and time points.

Participants

Participants are eligible if they are:

- Due to undergo primary (first-time) unilateral THR or TKR where the indication for surgery is degenerative joint pathology (not trauma).
- Aged 18 years and over.
- Classified as 'moderately inactive' or 'inactive' using on the General Practice Physical Activity Questionnaire (GPPAQ).[19]
- Have a Charlson Comorbidity Index (CCI) of ≥ 1 point.[20,21]

Participants are ineligible if they have:

- An absolute contraindication to exercise such as severe cardiovascular or pulmonary disease.
- Cognitive impairment defined as an Abbreviated Mental Test Score (AMTS)[22] of <8.
- A usual place of residence that is a care home.
- Already enrolled onto another trial investigating physical activity, exercise adherence or behavioural therapy interventions.
- An inability to read and/or comprehend English.
- No access to a working telephone.

Recruitment

Potential participants will be identified from UK NHS hospital trusts by the clinical team once they have been listed for THR or TKR. They will be asked whether they would like to know more about the PEP-TALK trial. There are two options for this: either they will be given a copy of the Participant Information Sheet (PIS) and asked to contact a member of the Clinical Research Network (CRN) research team who will provide more information or will be asked whether they are happy for a member of the CRN research team to contact them directly with more information about the trial. Verbal consent for the approach will be documented by a clinical team member in the medical notes.

Potential participants will be asked to read the PIS and asked to discuss their potential participation with anyone who they feel would provide useful advice such as friends, family member or carers. The number of people provided with the PIS will be recorded to monitor how many participants are assessed for initial eligibility and sent the PIS.

Eligible patients who agree to participate will provide their consent during the pre-operative assessment appointment. Participant's eligibility will then be verified by reviewing the medical notes and by interviewing the participant using the screening log, Charlson Comorbidity Index (CCI) and General Practice Physical Activity Questionnaire (GPPAQ) tools.

Written informed consent will be obtained prior to any trial-specific procedures being performed. The baseline case report form (CRF) will be completed at the pre-operative assessments once consent has been taken. After the TKR or THR, eligibility will be confirmed by the site research team member reviewing the post-operative notes.

For peer review only

Randomisation, Blinding and Allocation Concealment

Consented participants will be randomised (1:1) to the intervention group or the control group using the centralised computer randomisation service RRAMP (https://rramp.octru.ox.ac.uk) provided by the Oxford Clinical Trials Research Unit (OCTRU). This will either be undertaken directly by the site's research facilitator or by contacting the trial office over the telephone, who will access the system on their behalf. Randomisation will be undertaken using a minimisation algorithm to ensure balanced allocation of participants across the two treatment groups, stratified by:

- Hospital site
- Type of joint replacement (THR or TKR)
- CCI of 1-3 versus ≥4[20,21]

The minimisation algorithm will be seeded using simple randomisation and will have a probabilistic element introduced to ensure unpredictability of treatment assignment.

Baseline data will be collected during the pre-operative assessment appointment and prior to randomisation. Therefore, the central randomisation system will issue a screening identifier (ID). Once the participant has been randomised, the central randomisation system will provide a participant ID to be used on all subsequent data collection forms. Sites will be responsible for linking the screening ID to the participant ID, with all linking information remaining at sites. This will be double-checked across the CRFs to ensure that the correct paperwork has been provided to the correct participant.

Due to the nature of the intervention, participants and those delivering the rehabilitation will be aware of the treatment allocation. By virtue of this design, it is not possible to blind participants, physiotherapists or the site researchers.

Change to the randomisation allocation: An amendment was made to the randomisation ratio in the minimisation algorithm from 1:1 to 2:1 (Experimental Intervention: Usual Care) after 75 participants had been randomised. The expected allocation ratio at the end of trial is likely to approximately 1.5:1. This was done to ensure a greater number of people are allocated to the experimental intervention, which is a group-based intervention. As the experimental intervention was designed to have three or more people per group, early sites have found it difficult to consistently reach this level of participant numbers with the original 1:1 randomisation allocation. This change was made to reduce the risk of small numbers in the experimental group. The Trial Management Group (TMG) agreed this on 15th August 2019. The sample size was increased to 260 to account for this change. This was approved by the sponsor, Data and Safety Monitoring Committee (DSMC) and research ethics committee.

Intervention

Usual Care

This will be received by both control and intervention groups.

In the in-patient setting, all participants will be seen by a physiotherapist a minimum of daily on weekdays, and will follow their hospital's standard post-operative pathway. Average hospital length of stay for people after THR and TKR is five days.[1] Rehabilitation consists of gait re-education, exercises and advice regarding transferring from bed to chair, toileting and dressing. Advice on continuation of gait progression and activities of daily living are encouraged by the physiotherapy

For peer review only

team. Once considered medically fit and safe by the multidisciplinary team, patients are discharged. These follow current, routine, nationwide practice.[23]

All participants attend six-weekly, 30-minute group-based exercise classes within each hospital trust's physiotherapy department after THR or TKR. These groups consist of up to 12 people and commence within four weeks post-randomisation. The principles regarding prescription of group exercises to increase range of motion, strength and gait pattern are consistent. Whilst the rehabilitation of THR and TKR focuses on overall lower limb function, all participants following a THR focus on hip exercises, whereas those following a TKR focus on knee exercises. For this trial, exercise diaries will be provided and maintained by participants to monitor exercise performance and compliance within and outside the exercise groups for the six-week intervention period.

Experimental intervention

Participants randomised to the experimental group will receive the same in-hospital care and physiotherapy prior to hospital discharge. They will also receive the same six-weekly, group-based 30-minute exercise session. The only difference between the two groups is the addition of a 30-minute group-based (up to 12 people) behaviour change approach intervention (the PEP-TALK intervention) prior to the routine 30 minutes of exercise. Those randomised to the experimental group also receive three post-group telephone-based sessions.

The PEP-TALK intervention is theoretically-based within the cognitive-behavioural model of understanding. It uses evidence-based behaviour change techniques to target internal (cognitions and behaviours regarding physical activity) and external factors (social and environmental barriers). It specifically aims to target self-efficacy beliefs and fear-avoidant behaviours. During these sessions, through group discussion, participants and physiotherapists will be encouraged to develop a positive therapeutic alliance, where the physiotherapist will generate an environment of trust and belief around individual challenges participant's have, to support them to overcome these for sustained physical activity adoption. A treatment log will be completed by the physiotherapists to record the components of what is discussed across the group in each session.

The PEP-TALK intervention also includes a home-practice element. Participants will be supported with skills developed in the group, to work at home on challenges, barriers and facilitators to physical activity behaviour. The 'home-work' will include pacing and behaviour modification, goal-setting to the individual's health and social needs and techniques to challenge fear-avoidant behaviours. Education on exercise and the detrimental effects of physical inactivity will also be discussed. Participants will complete this with their home exercise plan which solely focuses on lower limb exercises rather than their behaviours and thoughts around physical activity.

The same trained physiotherapists who deliver the PEP-TALK intervention will, once the participant has completed their group sessions, deliver three, 20-minute telephone follow-up calls. These will occur two, four and six weeks after completing the group sessions. During these follow-up telephone calls, participant's goals will be reviewed, any barriers to the completion of these goals will be identified, and the physiotherapist will review any 'unhelpful' and 'helpful' thoughts or feelings towards physical activity which may have arisen since the last consultation. Each telephone call will close with the development of longer-term physical activity goal-setting and promotion of empowerment towards physical activity participation using the behavioural principles instilled during the group intervention. A log will be collected by the physiotherapist to record the components of what is discussed during the telephone calls.

Delivery

For peer review only

Both the PEP-TALK intervention and exercise groups will be delivered as 'rolling' programmes. This means that new participants can join the group as it runs rather than waiting for a new 'block' of sessions to start. This will allow greater flexibility in allocating participants to join group sessions, whilst also avoiding a delay between post-operative referral and starting the sessions. The flexible nature of the experimental intervention also makes this feasible, with no ordering of the content of intervention sessions which would preclude such a rolling programme.

Contamination

The physiotherapists who deliver the behaviour change sessions will be taught the skills required to deliver the experimental intervention. These physiotherapists will not be permitted to deliver the control exercise group intervention during the trial period (and vice versa). This approach mitigates the risk of contamination. Due to the interventions being delivered in an out-patient setting, there is a reduced risk of participants sharing their knowledge and experience of the interventions between the control and intervention groups, further minimising the risk of between-group contamination. Participants in the control group will not be permitted to join the experimental intervention group's exercise sessions (and vice versa). This will also reduce the risk of between-group contamination.

Co-Interventions

During the course of follow-up, participants may require further interventions as part of their recovery following surgery as per routine NHS practice. Further clinical interventions will be permitted for trial participants without the participant having to withdraw. If a participant receives additional treatment to the trial intervention, the details of the treatment received and the reasons will be collected.

Quality Assessment

The trial will be monitored and audited in accordance with the current approved protocol, good clinical practice,[24] relevant regulations and standard operating procedures (SOPs).

All designated physiotherapists who deliver the usual care group exercises will be taught about the standardised control intervention procedures, i.e. clarification on use of exercise diaries and treatment logs which are additional to usual care.

Designated physiotherapists delivering the behaviour change intervention will attend a one-day, face-to-face course where they will be taught the intervention and processes involved by a member of the PEP-TALK team who developed the intervention (BF, ZH, TS). In addition, to assess the fidelity to the trial intervention, a health psychologist (BF) will undertake a debriefing telephone call with each physiotherapist after they have delivered their first group session. This will allow the trainer to re-enforce any learning required, address any uncertainties and to identify and correct any variation in the treatment protocol. Finally, each physiotherapist who delivers either the PEP-TALK or control group intervention will be monitored during a site visit at their 3rd or 4th intervention session. The PEP-TALK intervention physiotherapist will be monitored by a health psychologist (BF) or physiotherapist with expertise in health psychology (ZH). Control group interventions will be monitored by a practicing physiotherapist (TS). Sessions will be monitored against the protocol to determine whether there are issues around fidelity, contamination across groups or adherence/compliance of participants. Where further training is required, this will be instigated following these visits. Further monitoring visits will be co-ordinated as required.

Assessments

For peer review only - http://bmjopen.bmj.com/site/about/guidelines.xhtml

Baseline Assessment

Baseline data will be collected prior to randomisation during the pre-operative assessment appointment, once consent has been obtained. This will include: gender, age, measured height and weight, CCI, self-reported presence and location of multi-site joint pain, co-morbidities determined from the medical notes, AMTS, employment status and occupation (when appropriate). Participants will also complete measures on: physical activity (University of California Los Angeles (UCLA) activity scale;[25] physical function (Lower Extremity Functional Scale (LEFS);[26] disease-specific function (Oxford Hip Score [27] or Oxford Knee Score [28]); pain (numerical rating scale for pain); self-efficacy (Generalized Self-Efficacy Scale [29]); fear of movement or kinesiophobia (Tampa Scale for Kinesiophobia [30]); psychological distress (Hospital Anxiety and Depression Scale (HADS) [31]); HRQoL (EQ-5D-5L [32]) and the health utilisation questionnaire.

Follow-up data will be collected six- and 12-months post-randomisation. Questionnaires will be sent to participants by post from the trial office and returned using a pre-addressed, pre-paid envelope. If participants have not responded within 14 days of posting, the trial team will attempt to telephone the participant on up to two occasions to remind them to complete the questionnaires. If required, a second postage of the questionnaires will be provided if requested by the participant during these follow-up telephone calls. If participants wish not to complete the questionnaires, they will be provided with the opportunity to complete the primary outcome and EQ-5D-5L questionnaires over the telephone. If these methods fail, the participant would be categorised as a non-responder for that time-point only.

In an effort to find evidence-based techniques to improve retention and recruitment to RCTs, a study within a trial (SWAT) will be undertaken. This will examine whether there is a difference in questionnaire response rate by printing the primary outcome measure on pink rather than white coloured paper at the six-month time-point. Results of this SWAT will be reported separately to the 'host' trial.

For peer review only

Outcome Measures

The data collection schedule is presented in **Table 2.**

Primary Outcome

- UCLA Activity Scale.[25] This is a reliable and valid self-reported tool to assess physical activity.[33,34] It assesses global activity levels with a grading system of one out of 10 points where one equates to "wholly inactive, dependent on others, and cannot leave residence" and 10 refers to "regularly participants in impact sports".[25]

Secondary Outcomes

- LEFS.[26] This is a valid and reliable measure of functional impairment in patients with lower extremity muscloskeletal condictions (Mehta et al, 2016).

- Oxford Hip Score (OHS) [27] or Oxford Knee Score (OKS).[28] Both measures have demonstrated good validity and reliability for people undergoing THR or TKR.[35]

- Numerical Rating Scale (NRS) for Pain. The NRS has been previously reported as reliable and valid to assess pain.[36]

- Generalized Self-Efficacy Scale.[29] This is a reliable and valid measure of self-efficacy.[37,38].

- The Tampa Scale for Kinesiophobia.[30] This is a valid and reliable measure of kinesiophobia.[39,40]

- HADS.[31] This has been shown as a reliable and valid measure of anxiety and depression.[41]

- Complications or adverse events (self-reported) which may include: wound or joint infection, joint dislocation, delayed hospital discharge (measured by length of stay), falls or musculoskeletal injuries and exacerbations of multi-site joint pain. Adverse events and serious adverse events will be reported as per the clinical trial unit's SOP.

- Health economic and health resource utilisation. We will collect HRQoL scores using the EQ-5D-5L.[33] Primary sources (i.e. participant log books) will be used to record the duration of the assessment visits, number and duration of scheduled group sessions, equipment, consumables, educational material, behaviour change intervention training time; number and duration of scheduled telephone calls. Volumes of health care resource use will be collected through patient self-reported questionnaires at follow-ups. The categories of NHS resource use that will be collected include treatment costs, outpatient visits, any related-inpatient admissions, rehabilitation visits and any treatment for recurrences and adverse events. Non-medical costs such as caregiving and employment-related information for those in employment at each follow-up will be collected.

Data Analysis

Sample Size

Originally, 250 participants (125 per arm) are required to detect a standardised effect size of 0.4 with 80% power and 5% (two-sided) significance, and allowing for 20% loss to follow-up. These calculations are based on the primary outcome, UCLA Activity Scale, at 12 months, assuming a baseline standard deviation of 2.5 and a between-group difference of one.[33] The minimally clinically important difference (MCID) has been reported as a within person difference of 0.92 points.[33]

The sample size was increased to 260 to account for the amendment in randomisation proceeedure from 1:1 to 2:1 group allocation, as described earlier.

Statistical Analysis

A detailed statistical analysis plan (SAP) will be drafted early in the trial and will be finalised prior to any primary outcome analysis. This will be reviewed and will receive input from the Trial Steering Committee (TSC) and the DSMC.

All analyses will be undertaken on the intent-to-treat population, i.e. patients will be analysed as they were randomised regardless of the treatment received. Sensitivity analyses will be undertaken on the per-protocol population to assess a range of potential biases that could have resulted from loss-to-

For peer review only

follow-up, protocol deviations and withdrawal (including mortality). Numerical and graphical summaries of all data will be presented including descriptions of missing data at each level.

Estimates of treatment effects will be reported with 95% confidence intervals. The primary outcome measure, UCLA Activity Scale at 12 months post-randomisation, will be analysed using a mixed-effects linear regression model adjusted for baseline activity and the stratification factors. Centre will be included as a random-effect to take account of their potential heterogeneity and type of operation and comorbidity index will be included as fixed-effects. The adjusted difference in the means with corresponding 95% confidence interval will be reported together with the mean and 95% confidence intervals for each treatment group. If not normally distributed, then transformation to normality will be considered. If this is not possible, non-parametric techniques (for example Mann-Whitney or Kruskal-Wallis Test) will be used with no adjustment. Consistency of any observed intervention effect will be explored using forest plots for various subgroups including site, type of operation (TKR and THR) and comorbidity index using interactions. No formal testing of interaction effects will be undertaken as the trial is not powered to detect these and the effect of the intervention is expected to be similar in the THR and TKR subgroups, although their baseline physical activity may be different, which will be taken into account in the analysis.

Similar methods will be used to analyse continuous secondary outcomes. Binary and categorical secondary outcomes, such as complications, will be tabulated to show frequencies and percentages in each arm. Chi-squared tests or logistic regression will be used to assess statistical significance.

Missing data will be minimised by careful data management. Missing data will be described with reasons given where available. The number and percentage of individuals in the missing category will be presented by treatment arm.

The nature and mechanism for missing variables and outcomes will be investigated, and if appropriate, multiple imputation will be used. Sensitivity analyses will be undertaken assessing the underlying missing data assumptions. Any imputation techniques will be fully described in the SAP. Subgroup analyses, complier average causal effect (CACE) analysis and mediation analyses will be undertaken after being fully specified in the SAP. Compliance with the intervention is defined as attending at least four of six group sessions with a minimum of three participants in the group for these sessions and receiving at least two of the three follow-up phone calls. A priori mediation analysis moderators will include self-efficacy, fear avoidance, psychological distress to compare the mediation pathways presented in the BeST intervention [42] to the PEP-TALK intervention.

All data collected on data collection forms will be used, since only essential data items will be collected. No data will be considered spurious in the analysis since all data will be checked and cleaned before analysis.

Health Economics

Respecting an *efficient trial design*, only if the data indicates clinical effectiveness of the experimental intervention (for the UCLA Activity Scale and LEFS), will additional funding be sought to analyse the health economic and utilisation data, to determine the cost-effectiveness of the intervention.

Data Management

For peer review only

All data will be processed according to the Data Protection Act 2018.[43] All documents will be stored safely in confidential conditions. Trial-specific documents, except for the signed consent form and follow-up contact details, will refer to the participant with a unique study participant number, not by name. Participant identifiable data will be stored separately from trial data. All trial data will be stored securely in offices or online in secure trial databases, only accessible by the central trial team in Oxford and authorised personnel.

Trial Status

The trial is funded for 36 months and commenced in August 2018. Recruitment is expected to be complete by April 2020 with the final follow-up visit for the final participant completed by April 2021. The trial will be completed by August 2021.

Site Locations

Orthopaedic services providing THR and TKRs in nine hospital trusts (eight recruiting sites) and health providers: (Norfolk and Norwich University Hospitals NHS Foundation Trust, Lewisham and Greenwich University Hospitals NHS Foundation Trust, Oxford University Hospital NHS Foundation Trust, City Hospitals Sunderland NHS Foundation Trust, Norwich Spire, Barts Health NHS Trust, North Middlesex University Hospital NHS Trust, St George's University Hospital NHS Foundation Trust (treatment site only), Epsom and St Helier University Hospitals NHS Foundation Trust (recruitment site only)).

Patient and Public Involvement

Patient involvement began during protocol development and continues throughout the trial. A patient-member will attend all TSC meetings. The same patient-member is a co-investigator, providing insights into the trial conduct, particularly on data collection processes, and will help interpret the findings to inform on the implications of the research during the trial's dissemination phase.

Ethics and Dissemination

Ethical approval was gained from the South Central (Oxford B) Research Ethics Committee (Approval Date: 23Oct2018; Reference Number: 18/SC/0423). The trial was prospectively registered (ISRCTN29770908). A DSMC and TSC was appointed to independently review the data on safety, protocol adherence and recruitment to the trial. Direct access will be granted to authorised representatives from the sponsor and host institution for monitoring and/or audit of the trial to ensure compliance with regulations. Annoymised data will be shared outside the research team when required. Researchers outside the trial team may formally request for a specific data set using a data request form, which will be part of the Data Management Plan (DMP). All such requests will need to be approved by the TMG.

Reporting of the trial will be consistent with the CONSORT 2010 Statement (patient reported outcomes and non-pharmcological interventions)[44] and Template for Intervention Description and Replication (TIDieR)[45] guidelines. A summary of the results and trial materials will be made available

For peer review only

via the trial website on completion of the trial. We will submit the final report to a peer-reviewed academic journal.

DISCUSSION

This paper presents the research protocol for the PEP-TALK trial. Following a THR and TKR, only 50% of people reach World Health Organization recommended levels of physical activity.[15,46,47] Those who are least likely to meet these levels are people with a higher body mass index and with comorbidities.[48] These patients have the most to gain from being more physically active. If this trial's experimental intervention is shown to be effective, this intervention could have a significant and sustained impact on improving the management of comorbidities such as diabetes, cardiovascular diseases, depression and hypertension for over 103,000 people annually in England and Wales.[1] It is proposed that, in such an instance, this should be considered for implementation in healthcare services. This could help address the global challenge which multi-morbidities and an ageing population are expected to have on health and social care services.

DECLARATIONS AND ACKNOWLEDGEMENTS

Contributors: TS, BF, SJD, SL, AO, CH, VB, SP, SA researched the topic and devised the study. TS, SP, AO, BF, SJD, CH, SA, VB, MEP, SL provided the first draft of the manuscript. AO, SD provided statistical oversight. TS, SP, BF, AO, SJD, CH, VB, MEP, SA, SL contributed equally to manuscript preparation. TS acts a guarantor.

Funding: The research is supported by the National Institute for Health Research (NIHR) Research for Patient Benefit grant (PB-PG-1216-20008). The views expressed are those of the author(s) and not necessarily those of the NHS, the NIHR or the Department of Health and Social Care.

Competing interests: None declared.

Disclaimer: TS is supported by the National Institute for Health Research (NIHR) Oxford Biomedical Research Centre (BRC). The views expressed are those of the author(s) and not necessarily those of the NHS, the NIHR or the Department of Health and Social Care.

Ethics approval: Ethical approval was gained from the South Central (Oxford B) Research Ethics Committee (Approval Date: 23Oct2018; Reference Number: 18/SC/0423).

Trial Sponsor: University of Oxford (Clinical Trials and Research Governance Team. Joint Research Office, 1st floor, Boundary Brook House, Churchill Drive, Headington, Oxford OX3 7GB. Email: ctrg@admin.ox.ac.uk. The views expressed are those of the author(s) and not necessarily those of the sponsor.

Provenance and peer review: Not commissioned; externally peer reviewed.

PEP-TALK Collaborators: Dr Zara Hansen (ZH) (University of Oxford), Professor Karen Barker (Principal Investigator - Oxford University Hospital NHS Foundation Trust (OUH)**,** Mr Ian Smith (Principal Investigator **-** Lewisham and Greenwich University Hospitals NHS Foundation Trust), Professor Iain McNamara (Principal Investigator (Norfolk and Norwich University Hospitals NHS Foundation Trust (NNUH) & Spire Norwich), Mr Michael Dunn (Principal Investigator - St George's University Hospital

For peer review only

NHS Foundation Trust), Mrs Dawn Lockey (Principal Investigator - City Hospitals Sunderland NHS Foundation Trust), Mr Sonny Driver (Principal Investigator – North Middlesex University Hospital NHS Trust), Mrs Jamila Kassam (Barts Health NHS Trust), Mr Peter Penny (Norwich Spire, Norwich), Mrs Celia Woodhouse, Mrs Tracey Potter and Mrs Helena Daniell (NNUH), Mr Alex Herring and Mrs Yan Cunningham (City Hospitals Sunderland NHS Foundation Trust), Irrum Afzal (South West London Elective Orthopaedic Centre), Mr Maninderpal Matharu (Barts Health NHS Trust) and Mrs Tamsin Hughes, Ms Erin Hannink and Mrs Michelle Moynihan (OUH).

Oversight Committee Membership: TSC Members: Professor David Deehan (Newcastle University), Dr Emma Godfrey (Kings College London), Dr Neil Artz (University of the West of England), Mr Steve Algar (PPI representative). **DSMC Members:** Dr Lindsey Smith (University of the West of England & Weston Area Health NHS Trust), Dr Dipesh Mistry (University of Warwick), Mr Paul Baker (South Tees Hospital NHS Foundation Trust)

Provenance and peer review: Not commissioned; externally peer reviewed.

Patient consent for publication: Not required.

Word count: 4360

FIGURE AND TABLE LEGENDS

Figure 1: Study flow chart

Table 1: Data collection schedule

Table 2: PEP-TALK trial objectives, outcome measures and measurement time-points

REFERENCES

1. National Joint Registry. 12th Annual Report. Accessed 15.09.2016. Available at: http://www.njrcentre.org.uk/njrcentre/Portals/0/Documents/England/Reports/12th%20annual%20eport/NJR%20Online%20Annual%20Report%202015.pdf

2. Dailiana ZH, Papakostidou I, Varitimidis S, Liaropoulos L, Zintzaras E, Karachalios T, Michelinakis E, Malizos KN. Patient-reported quality of life after primary major joint arthroplasty: a prospective comparison of hip and knee arthroplasty. BMC Musculoskelet Disord. 2015 Nov 26;16:366.

3. Papakostidou I, Dailiana ZH, Papapolychroniou T, Liaropoulos L, Zintzaras E, Karachalios TS, Malizos KN. Factors affecting the quality of life after total knee arthroplasties: a prospective study. BMC Musculoskelet Disord. 2012 Jun 29;13:116.

4. Withers TM, Lister S, Sackley C, Clark A, Smith TO. Is there a difference in physical activity levels in patients before and up to one year after unilateral total hip replacement? A systematic review and meta-analysis. Clin Rehabil. 2016: In Press.

For peer review only

5. Smith TO, Hilton G, Mansfield M, Mann CJV, Sackley CM. Does physical activity change following hip and knee arthroplasty? An analysis of data from the Osteoarthritis Initiative. Physiotherapy. 2018; In Press.

6. Smith TO, Latham S, Maskrey V, Blyth A. Patients' perceptions of physical activity before and after joint replacement: a systematic review with meta-ethnographic analysis. Postgrad Med J. 2015 Sep;91(1079):483-91.

7. Inacio MC, Pratt NL, Roughead EE, Graves SE. Comparing co-morbidities in total joint arthroplasty patients using the RxRisk-V, Elixhauser, and Charlson Measures: a cross-sectional evaluation. BMC Musculoskelet Disord. 2015 Dec 10;16:385.

8. Peter WF, Dekker J, Tilbury C, Tordoir RL, Verdegaal SH, Onstenk R, Bénard MR, Vehmeijer SB, Fiocco M, Vermeulen HM, van der Linden-van der Zwaag HM, Nelissen RG, Vliet Vlieland TP. The association between comorbidities and pain, physical function and quality of life following hip and knee arthroplasty. Rheumatol Int. 2015 Jul;35(7):1233-41.

9. Smith E, Hoy DG, Cross M, Vos T, Naghavi M, Buchbinder R, Woolf AD, March L. PICO. The global burden of other musculoskeletal disorders: estimates from the Global Burden of Disease 2010 study. Ann Rheum Dis. 2014 Aug;73(8):1462-9.

10. Sampson UK, Fowkes FG, McDermott MM, Criqui MH, Aboyans V, Norman PE, Forouzanfar MH, Naghavi M, Song Y, Harrell FE Jr, Denenberg JO, Mensah GA, Ezzati M, Murray C. Global and regional burden of death and disability from peripheral artery disease: 21 world regions, 1990 to 2010. Glob Heart. 2014 Mar;9(1):145-158.e21.

11. Lee CD, Folsom AR, Blair SN. Physical activity and stroke risk: a meta-analysis. Stroke. 2003 Oct;34(10):2475-81.

12. Laaksonen DE, Lindström J, Lakka TA, Eriksson JG, Niskanen L, Wikström K, Aunola S, Keinänen-Kiukaanniemi S, Laakso M, Valle TT, Ilanne-Parikka P, Louheranta A, Hämäläinen H, Rastas M, Salminen V, Cepaitis Z, Hakumäki M, Kaikkonen H, Härkönen P, Sundvall J, Tuomilehto J, Uusitupa M; Finnish diabetes prevention study. Physical activity in the prevention of type 2 diabetes: the Finnish diabetes prevention study. Diabetes. 2005 Jan;54(1):158-65.

13. Lee IM. Physical activity and cancer prevention--data from epidemiologic studies. Med Sci Sports Exerc. 2003 Nov;35(11):1823-7.

14. Nocon M, Hiemann T, Müller-Riemenschneider F, Thalau F, Roll S, Willich SN. Association of physical activity with all-cause and cardiovascular mortality: a systematic review and meta-analysis. Eur J Cardiovasc Prev Rehabil. 2008 Jun;15(3):239-46.

15. Kersten RF, Stevens M, van Raay JJ, Bulstra SK, van den Akker-Scheek I. Habitual physical activity after total knee replacement. Phys Ther. 2012 Sep;92(9):1109-16.

16. French DP, Olander EK, Chisholm A, Mc Sharry J. Which behaviour change techniques are most effective at increasing older adults' self-efficacy and physical activity behaviour? A systematic review. Ann Behav Med. 2014 Oct;48(2):225-34.

For peer review only

17. Devereux-Fitzgerald A, Powell R, Dewhurst A, French DP. The acceptability of physical activity interventions to older adults: A systematic review and meta-synthesis. Soc Sci Med. 2016 Jun;158:14-23.

18. Friederichs SA, Oenema A, Bolman C, Lechner L. Long term effects of self-determination theory and motivational interviewing in a web-based physical activity intervention: randomized controlled trial. Int J Behav Nutr Phys Act. 2015 Aug 18;12:101.

19. Department of Health. 2013. General Practice Physical Activity Questionnaire. Accessed on 21.02.2017. Available at: https://www.gov.uk/government/publications/general-practice-physical-activity-questionnaire-gppaq

20. Charlson ME, Pompei P, Ales KL, MacKenzie CR. A new method of classifying prognostic comorbidity in longitudinal studies: development and validation. J Chronic Dis. 1987;40(5):373-83.

21. Charlson ME, Charlson RE, Briggs W, Hollenberg J. Can disease management target patients most likely to generate high costs? The impact of comorbidity. J Gen Intern Med. 2007; 22: 464-469.

22. Hodkinson HM. Evaluation of a mental test score for assessment of mental impairment in the elderly. Age Ageing 1972;1: 233–8.

23. Briggs TWR. 2012. Getting it right first time. Improving the quality of orthopaedic care within the National Health Service in England. Accessed 06.10.2016. Available at: http://www.gettingitrightfirsttime.com/downloads/briggsreporta4_fin.pdf

24. International Conference on Harmonisation Good Clinical Practice (ICH GCP). International Conference on Harmonisation Good Clinical Practice Recommendations. Accessed: 08 March 2018. Available at: https://www.ich.org/fileadmin/Public_Web_Site/ICH_Products/Guidelines/Efficacy/E6/E6_R1_Guideline.pdf

25. Zahiri CA, Schmalzried TP, Szuszczewicz ES, Amstutz HC. Assessing activity in joint replacement patients. J Arthroplasty. 1998;13:890–895

26. Binkley JM, Stratford OW, Lott SA, Riddle DL. The Lower Extremity Functional Scale (LEFS): Scale development, measurement properties, and clinical application. Physical Therapy 1999;79:371-383

27. Dawson J, Fitzpatrick R, Carr A, Murray D. Questionnaire on the perceptions of patients about total hip replacement. J Bone Joint Surg Br. 1996 Mar;78(2):185-90.

28. Dawson J, Fitzpatrick R, Murray D, Carr A. Questionnaire on the perceptions of patients about total knee replacement. J Bone Joint Surg Br. 1998 Jan;80(1):63-9.

29. Schwarzer R, Jerusalem M. Generalized Self-Efficacy scale. In J. Weinman, S. Wright, & M. Johnston, Measures in health psychology: A user's portfolio. Causal and control beliefs 1995, pp. 35-37.

30. Miller RP, Kori S, Todd D. The Tampa Scale: a measure of kinesiophobia. Clin J Pain 1991;7(1):51–52.

For peer review only

31. Zigmond AS, Snaith RP. The hospital anxiety and depression scale. Acta Psychiatrica Scandinavica. 1983; 67: 361–370.

32. EuroQol Group E (1990) EuroQol—a new facility for the measurement of health-related quality of life. Health Policy 16: 199–208.

33. SooHoo NF, Li Z, Chenok KE, Bozic KJ. Responsiveness of patient reported outcome measures in total joint arthroplasty patients. J Arthroplasty. 2015 Feb;30(2):176-91.

34. Naal FD, Impellizzeri FM, Leunig M. Which is the best activity rating scale for patients undergoing total joint arthroplasty? Clin Orthop Relat Res. 2009 Apr;467(4):958-65.

35. Harris K, Dawson J, Gibbons E, Lim CR, Beard DJ, Fitzpatrick R, Price AJ. Systematic review of measurement properties of patient-reported outcome measures used in patients undergoing hip and knee arthroplasty. Patient Relat Outcome Meas. 2016 Jul 25;7:101-8. doi: 10.2147/PROM.S97774.

36. Ferreira-Valente MA, Pais-Ribeiro JL, Jensen MP. Validity of four pain intensity rating scales. Pain. 2011 Oct;152(10):2399-404.

37. Bonsaksen T, Kottorp A, Gay C, Fagermoen MS, Lerdal A. Rasch analysis of the General Self-Efficacy Scale in a sample of persons with morbid obesity. Health Qual Life Outcomes. 2013 Nov 25;11:202. doi: 10.1186/1477-7525-11-202.

38. Barlow JH, Williams B, Wright C. The Generalized Self-Efficacy Scale in people with arthritis. Arthritis Care Res. 1996 Jun;9(3):189-96.

39. Lundberg MKE, Styf J, Carlsson SG. A psychometric evaluation of the Tampa Scale for Kinesiophobia – from a physiotherapeutic perspective. Physiotherapy Theory and Practice. 2004;20(2):121–133.

40. Bunketorp L, Carlsson J, Kowalski J, Stener-Victorin E. Evaluating the reliability of multi-item scales: a non-parametric approach to the ordered categorical structure of data collected with the Swedish version of the Tampa Scale for Kinesiophobia and the Self-Efficacy Scale. J Rehabil Med. 2005 Sep;37(5):330-4

41. Bjelland I, Dahl AA, Haug TT, Neckelmann D. The validity of the Hospital Anxiety and Depression Scale: an updated literature review. J Psychosom Res. 2002;52(2):69–77

42. Lamb SE, Lall R, Hansen Z, Castelnuovo E, Withers EJ, Nichols V, Griffiths F, Potter R, Szczepura A, Underwood M; BeST trial group. A multicentred randomised controlled trial of a primary care-based cognitive behavioural programme for low back pain. The Back Skills Training (BeST) trial. Health Technol Assess. 2010 Aug;14(41):1-253, iii-iv.

43. Data Protection Act. 2018. Accessed: 30 September 2019. Available at: http://www.legislation.gov.uk/ukpga/2018/12/contents/enacted

44. Schulz KF , Altman DG , Moher D , et al. CONSORT 2010 statement: updated guidelines for reporting parallel group randomised trials. BMC Med 2010;8:18

45. Hoffmann TC , Glasziou PP, Boutron I, et al. Better reporting of interventions: template for intervention description and replication (TIDieR) checklist and guide. BMJ 2014;348:g1687

For peer review only

46. Groen JW, Stevens M, Kersten RF, Reininga IH, van den Akker-Scheek I. After total knee arthroplasty, many people are not active enough to maintain their health and fitness: an observational study. J Physiother. 2012;58(2):113-6.

47. Wagenmakers R, Stevens M, Zijlstra W, Jacobs ML, van den Akker-Scheek I, Groothoff JW, Bulstra SK. Habitual physical activity behavior of patients after primary total hip arthroplasty. Phys Ther. 2008 Sep;88(9):1039-48.

48. Paxton EW, Torres A, Love RM, Barber TC, Sheth DS, Inacio MC. Total joint replacement: A multiple risk factor analysis of physical activity level 1-2 years postoperatively. Acta Orthop. 2016 Jul;87 Suppl 1:44-9.

For peer review only

Table 1: PEP-TALK trial objectives, outcome measures and measurement time-points

Objectives	Outcome Measures	Time-points
**Primary Objective**		
To compare physical activity participation at 12 months post randomisation between people who receive a group exercise and behaviour change intervention versus group exercise alone following THR or TKR.	University of California, Los Angeles (UCLA) activity scale	Baseline; 6 months; 12 months (primary time-point)
**Secondary Objectives**		
To compare the functional outcome between people who receive a group exercise and behaviour-change intervention versus group exercise alone following THR or TKR.	Lower Extremity Functional Scale (LEFS)	Baseline; 6 months; 12 months
To compare the health-related quality of life between people who receive a group exercise and behaviour-change intervention versus group exercise alone following THR or TKR.	Oxford Hip Score (OHS) [35] or Oxford Knee Score (OKS)	Baseline; 6 months; 12 months
To compare the perceived level of pain between people who receive a group exercise and behaviour-change intervention versus group exercise alone following THR or TKR.	Numerical Rating Scale (NRS) for Pain	Baseline; 6 months; 12 months
To compare participant's self-efficacy between those who receive a group exercise and behaviour-change intervention versus group exercise alone following THR or TKR.	Generalized Self-Efficacy Scale (GSES)	Baseline; 6 months; 12 months
To compare participant's fear avoidance to movement between those who receive a group exercise and behaviour-change intervention versus group exercise alone following THR or TKR.	The Tampa Scale for Kinesiophobia	Baseline; 6 months; 12 months
To compare participant's psychological distress (anxiety and depression) between those who receive a group exercise and behaviour-change intervention versus group exercise alone following THR or TKR.	Hospital Anxiety and Depression Scale (HADS)	Baseline; 6 months; 12 months
To compare participant's complications and adverse events between those who receive a group exercise and behaviour-change intervention versus group exercise alone following THR or TKR.	Complications or adverse events recorded in the CRFs	6 months; 12 months
To collect cost-effectiveness data (health resource utilisation; direct and indirect costs) of a group exercise and a behaviour-change intervention versus group exercise alone following THR or TKR.	EQ-5D-5L; Bespoke health care resource self-reported questionnaire	6 months; 12 months

For peer review only

Table 2: Data collection schedule

**Data**	**Time-points and Mode of Data Collection**			
	**Baseline**	**Intervention Period**	**Month 6 Post-Randomisation**	**Month 12 Post-Randomisation**
	**Face-to-Face**	**Face-to-Face**	**Postal/Online**	**Postal/Online**
Age (years)				
Gender				
Weight (kg)				
Height (cm)				
Admission Date				
Operative procedure (THR or TKR)				
Side of Joint Replacement				
Duration of hip or knee symptoms				
Presence and location of multi-joint pain				
ASA grade				
AMTS				
List of medical co-morbidities				
Physiotherapist exercise class log				
Patient in-session and home exercise diary				
Behaviour change intervention log (group and telephone) (physiotherapist completed)				
Charlson Comorbidity Score				
Employment status and current occupation (when appropriate)				
UCLA Activity Score				
Lower Extremity Functional Scale				
Oxford Hip or Knee Score				
Numerical Rating Scale – Pain				
Generalized Self-Efficacy Scale				
Tampa Scale for Kinesiophobia				
Hospital Anxiety and Depression Scale				
EQ-5D-5L				
Health economic/Health utilisation questionnaire				
Complications and adverse events				

Assessment intervals following randomisation; each follow-up interval +/- 1 month

AMTS – Abbreviated Mental Test Score; ASA – American Society of Anaesthesiologists classification; cm – centimetres; kg – kilograms; THR – total hip replacement; TKR – total knee replacement; UCLA – University College Los Angeles.

For peer review only

Figure 1: Study flow chart

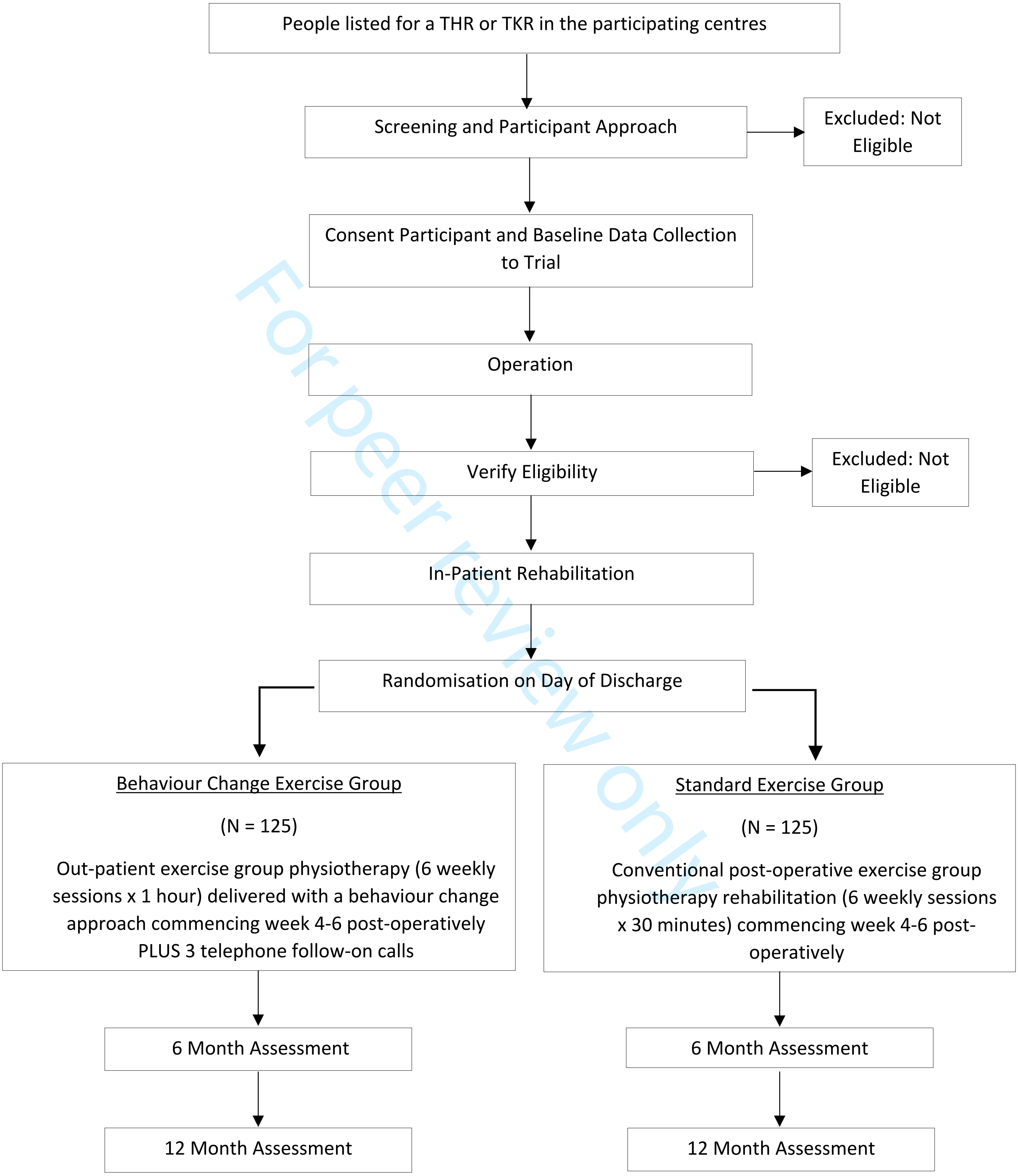

For peer review only

SPIRIT

STANDARD PROTOCOL ITEMS: RECOMMENDATIONS FOR INTERVENTIONAL TRIALS

SPIRIT 2013 Checklist: Recommended items to address in a clinical trial protocol and related documents*

Section/item	Item No	Description	Location in Text
**Administrative information**			
Title	1	Descriptive title identifying the study design, population, interventions, and, if applicable, trial acronym	Title
Trial registration	2a	Trial identifier and registry name. If not yet registered, name of intended registry	Abstract
	2b	All items from the World Health Organization Trial Registration Data Set	Abstract to ID
Protocol version	3	Date and version identifier	Abstract page
Funding	4	Sources and types of financial, material, and other support	Abstract page & Declarations
Roles and responsibilities	5a	Names, affiliations, and roles of protocol contributors	Title Page & Declarations
	5b	Name and contact information for the trial sponsor	Declarations
	5c	Role of study sponsor and funders, if any, in study design; collection, management, analysis, and interpretation of data; writing of the report; and the decision to submit the report for publication, including whether they will have ultimate authority over any of these activities	Declarations

For peer review only

	5d	Composition, roles, and responsibilities of the coordinating centre, steering committee, endpoint adjudication committee, data management team, and other individuals or groups overseeing the trial, if applicable (see Item 21a for data monitoring committee)	Data Management and Ethics and Dissemination
**Introduction**			
Background and rationale	6a	Description of research question and justification for undertaking the trial, including summary of relevant studies (published and unpublished) examining benefits and harms for each intervention	Background, Para 5
	6b	Explanation for choice of comparators	BACkground Para 3-4; Methods, Interventions
Objectives	7	Specific objectives or hypotheses	Background, Para 4
Trial design	8	Description of trial design including type of trial (eg, parallel group, crossover, factorial, single group), allocation ratio, and framework (eg, superiority, equivalence, noninferiority, exploratory)	Methods, Trial Design
**Methods: Participants, interventions, and outcomes**			
Study setting	9	Description of study settings (eg, community clinic, academic hospital) and list of countries where data will be collected. Reference to where list of study sites can be obtained	Methods, Site Locations & Declarations
Eligibility criteria	10	Inclusion and exclusion criteria for participants. If applicable, eligibility criteria for study centres and individuals who will perform the interventions (eg, surgeons, psychotherapists)	Methods, Participants

For peer review only

Interventions	11a	Interventions for each group with sufficient detail to allow replication, including how and when they will be administered	Methods, Interventions
	11b	Criteria for discontinuing or modifying allocated interventions for a given trial participant (eg, drug dose change in response to harms, participant request, or improving/worsening disease)	Methods, Contamination
	11c	Strategies to improve adherence to intervention protocols, and any procedures for monitoring adherence (eg, drug tablet return, laboratory tests)	Methods, Quality Assessment
	11d	Relevant concomitant care and interventions that are permitted or prohibited during the trial	Methods, Cointerventions
Outcomes	12	Primary, secondary, and other outcomes, including the specific measurement variable (eg, systolic blood pressure), analysis metric (eg, change from baseline, final value, time to event), method of aggregation (eg, median, proportion), and time point for each outcome. Explanation of the clinical relevance of chosen efficacy and harm outcomes is strongly recommended	Methods, Outcome Measures & Table 2
Participant timeline	13	Time schedule of enrolment, interventions (including any run-ins and washouts), assessments, and visits for participants. A schematic diagram is highly recommended (see Figure)	Figure 1
Sample size	14	Estimated number of participants needed to achieve study objectives and how it was determined, including clinical and statistical assumptions supporting any sample size calculations	Data Analysis, Sample Size
Recruitment	15	Strategies for achieving adequate participant enrolment to reach target sample size	Methods, Trial Status, Site Locations
**Methods: Assignment of interventions (for controlled trials)**			
Allocation:			

Sequence generation	16a	Method of generating the allocation sequence (eg, computer-generated random numbers), and list of any factors for stratification. To reduce predictability of a random sequence, details of any planned restriction (eg, blocking) should be provided in a separate document that is unavailable to those who enrol participants or assign interventions	Methods, Randomisation, Blinding and Allocation
Allocation concealment mechanism	16b	Mechanism of implementing the allocation sequence (eg, central telephone; sequentially numbered, opaque, sealed envelopes), describing any steps to conceal the sequence until interventions are assigned	Methods, Randomisation, Blinding and Allocation
Implementation	16c	Who will generate the allocation sequence, who will enrol participants, and who will assign participants to interventions	Methods, Randomisation, Blinding and Allocation
Blinding (masking)	17a	Who will be blinded after assignment to interventions (eg, trial participants, care providers, outcome assessors, data analysts), and how	Methods, Randomisation, Blinding and Allocation
	17b	If blinded, circumstances under which unblinding is permissible, and procedure for revealing a participant's allocated intervention during the trial	Methods, Randomisation, Blinding and Allocation
**Methods: Data collection, management, and analysis**			
Data collection methods	18a	Plans for assessment and collection of outcome, baseline, and other trial data, including any related processes to promote data quality (eg, duplicate measurements, training of assessors) and a description of study instruments (eg, questionnaires, laboratory tests) along with their reliability and validity, if known. Reference to where data collection forms can be found, if not in the protocol	Methods, Outcome Measures & Table 2

For peer review only - http://bmjopen.bmj.com/site/about/guidelines.xhtml

	18b	Plans to promote participant retention and complete follow-up, including list of any outcome data to be collected for participants who discontinue or deviate from intervention protocols	Methods, Assessments
Data management	19	Plans for data entry, coding, security, and storage, including any related processes to promote data quality (eg, double data entry; range checks for data values). Reference to where details of data management procedures can be found, if not in the protocol	Methods, Assessment & Data Management
Statistical methods	20a	Statistical methods for analysing primary and secondary outcomes. Reference to where other details of the statistical analysis plan can be found, if not in the protocol	Data Analysis
	20b	Methods for any additional analyses (eg, subgroup and adjusted analyses)	Data Analysis
	20c	Definition of analysis population relating to protocol non-adherence (eg, as randomised analysis), and any statistical methods to handle missing data (eg, multiple imputation)	Data Analysis
**Methods: Monitoring**			
Data monitoring	21a	Composition of data monitoring committee (DMC); summary of its role and reporting structure; statement of whether it is independent from the sponsor and competing interests; and reference to where further details about its charter can be found, if not in the protocol. Alternatively, an explanation of why a DMC is not needed	Data Management & Ethics and Dissemination & Declarations
	21b	Description of any interim analyses and stopping guidelines, including who will have access to these interim results and make the final decision to terminate the trial	Data Management & Ethics and Dissemination
Harms	22	Plans for collecting, assessing, reporting, and managing solicited and spontaneously reported adverse events and other unintended effects of trial interventions or trial conduct	Data Management

For peer review only - http://bmjopen.bmj.com/site/about/guidelines.xhtml

Auditing	23	Frequency and procedures for auditing trial conduct, if any, and whether the process will be independent from investigators and the sponsor	Quality Assessment
**Ethics and dissemination**			
Research ethics approval	24	Plans for seeking research ethics committee/institutional review board (REC/IRB) approval	Declarations
Protocol amendments	25	Plans for communicating important protocol modifications (eg, changes to eligibility criteria, outcomes, analyses) to relevant parties (eg, investigators, REC/IRBs, trial participants, trial registries, journals, regulators)	Ethics and Dissemination
Consent or assent	26a	Who will obtain informed consent or assent from potential trial participants or authorised surrogates, and how (see Item 32)	Methods, Recruitment
	26b	Additional consent provisions for collection and use of participant data and biological specimens in ancillary studies, if applicable	Not applicable
Confidentiality	27	How personal information about potential and enrolled participants will be collected, shared, and maintained in order to protect confidentiality before, during, and after the trial	Data Management
Declaration of interests	28	Financial and other competing interests for principal investigators for the overall trial and each study site	Declarations
Access to data	29	Statement of who will have access to the final trial dataset, and disclosure of contractual agreements that limit such access for investigators	Data Management
Ancillary and post-trial care	30	Provisions, if any, for ancillary and post-trial care, and for compensation to those who suffer harm from trial participation	Not applicable
Dissemination policy	31a	Plans for investigators and sponsor to communicate trial results to participants, healthcare professionals, the public, and other relevant groups (eg, via publication, reporting in results databases, or other data sharing arrangements), including any publication restrictions	Ethics and Dissemination

For peer review only - http://bmjopen.bmj.com/site/about/guidelines.xhtml

	31b	Authorship eligibility guidelines and any intended use of professional writers	Not applicable
	31c	Plans, if any, for granting public access to the full protocol, participant-level dataset, and statistical code	Ethics and Dissemination
**Appendices**			
Informed consent materials	32	Model consent form and other related documentation given to participants and authorised surrogates	Available on request
Biological specimens	33	Plans for collection, laboratory evaluation, and storage of biological specimens for genetic or molecular analysis in the current trial and for future use in ancillary studies, if applicable	Not applicable.

*It is strongly recommended that this checklist be read in conjunction with the SPIRIT 2013 Explanation & Elaboration for important clarification on the items. Amendments to the protocol should be tracked and dated. The SPIRIT checklist is copyrighted by the SPIRIT Group under the Creative Commons "Attribution-NonCommercial-NoDerivs 3.0 Unported" license.

For peer review only

BMJ Open

Behaviour change physiotherapy intervention to increase physical activity following hip and knee replacement (PEP-TALK): study protocol for a pragmatic randomised controlled trial

Journal:	*BMJ Open*
Manuscript ID	bmjopen-2019-035014.R1
Article Type:	Protocol
Date Submitted by the Author:	20-Mar-2020
Complete List of Authors:	Smith, Toby O.; University of Oxford , Nuffield Department of Orthopaedics, Rheumatology and Musculoskeletal Sciences; University of East Anglia, Faculty of Medicine and Health Sciences<br>Parsons, Scott; University of Oxford, Nuffield Department of Orthopaedics, Rheumatology and Musculoskeletal Disorders<br>Fordham, Beth; University of Oxford , NDORMS<br>Ooms, Alexander; University of Oxford, Nuffield Department of Orthopaedics, Rheumatology and Musculoskeletal Disorders<br>Dutton, Susan; University of Oxford, CSM<br>Hing, Caroline; University of London St George's Molecular and Clinical Sciences Research Institute<br>Barber, Vicki; University of Oxford Nuffield Department of Orthopaedics Rheumatology and Musculoskeletal Sciences, Oxford Clinical Trials Unit<br>Png, May ee; University of Oxford, NDORMS<br>Lamb, Sarah; University of Oxford, Ndorms; University of Exeter, College of Medicine and Health Sciences
**Primary Subject Heading**:	Rheumatology
Secondary Subject Heading:	Surgery
Keywords:	Adult orthopaedics < ORTHOPAEDIC & TRAUMA SURGERY, Hip < ORTHOPAEDIC & TRAUMA SURGERY, Knee < ORTHOPAEDIC & TRAUMA SURGERY

SCHOLARONE™ Manuscripts

I, the Submitting Author has the right to grant and does grant on behalf of all authors of the Work (as defined in the below author licence), an exclusive licence and/or a non-exclusive licence for contributions from authors who are: i) UK Crown employees; ii) where BMJ has agreed a CC-BY licence shall apply, and/or iii) in accordance with the terms applicable for US Federal Government officers or employees acting as part of their official duties; on a worldwide, perpetual, irrevocable, royalty-free basis to BMJ Publishing Group Ltd ("BMJ") its licensees and where the relevant Journal is co-owned by BMJ to the co-owners of the Journal, to publish the Work in this journal and any other BMJ products and to exploit all rights, as set out in our licence.

The Submitting Author accepts and understands that any supply made under these terms is made by BMJ to the Submitting Author unless you are acting as an employee on behalf of your employer or a postgraduate student of an affiliated institution which is paying any applicable article publishing charge ("APC") for Open Access articles. Where the Submitting Author wishes to make the Work available on an Open Access basis (and intends to pay the relevant APC), the terms of reuse of such Open Access shall be governed by a Creative Commons licence – details of these licences and which Creative Commons licence will apply to this Work are set out in our licence referred to above.

Other than as permitted in any relevant BMJ Author's Self Archiving Policies, I confirm this Work has not been accepted for publication elsewhere, is not being considered for publication elsewhere and does not duplicate material already published. I confirm all authors consent to publication of this Work and authorise the granting of this licence.

For peer review only

BMJ OPEN

TITLE PAGE

Title: Behaviour change physiotherapy intervention to increase physical activity following hip and knee replacement (PEP-TALK): study protocol for a pragmatic randomised controlled trial

Authors: Smith TO,[1,2] Parsons SR,[1] Fordham B,[1] Ooms AG,[1,3] Dutton SJ,[1,3] Hing CB,[4] Barber VS,[1,3] Png ME,[1,3] Lamb SE[1,5] on behalf of the PEP-TALK Trial Collaborators.

Affiliations

1. Nuffield Department of Rheumatology, Orthopaedics and Musculoskeletal Sciences, University of Oxford, Botnar Research Centre, Windmill Road, Oxford, UK
2. Faculty of Medicine and Health Sciences, University of East Anglia, Norwich, UK
3. Oxford Clinical Trials Research Unit (OCTRU), University of Oxford, Windmill Road, Oxford, UK
4. Department of Trauma and Orthopaedics, St George's Hospital, St George's University Hospitals NHS Foundation Trust, Tooting, London, UK
5. College of Medicine and Health Sciences, University of Exeter, Exeter, UK

Corresponding Author: Dr Toby Smith, Nuffield Department of Rheumatology, Orthopaedics and Musculoskeletal Sciences, University of Oxford, Botnar Research Centre, Windmill Road, Oxford, UK. Email: toby.smith@ndorms.ox.ac.uk

Word Count: 4674

For peer review only

ABSTRACT

INTRODUCTION: Whilst total hip (THR) and knee replacements (TKR) successfully reduce pain associated with chronic joint pathology, this infrequently translates into increased physical activity. This is a challenge given that over 50% of individuals who undergo these operations are physically inactive and have medical comorbidities such as hypertension, heart disease, diabetes and depression. The impact of these diseases can be reduced with physical activity. This trial aims to investigate the effectiveness of a behaviour change physiotherapy intervention to increase physical activity compared to usual rehabilitation after THR or TKR.

METHODS AND ANALYSIS: The PEP-TALK trial is a multi-centre, open-labelled, pragmatic randomised controlled trial. 260 adults who are scheduled to undergo a primary unilateral THR or TKR and are moderately inactive or inactive, with comorbidities, will be recruited across eight sites in England. They will be randomised post-surgery, prior to hospital discharge, to either six, 30-minute weekly group-based exercise sessions(control), or the same six-weekly, group-based, exercise sessions each preceded by a 30-minute cognitive behaviour approach discussion group. Participants will be followed-up to 12 months by postal questionnaire. The primary outcome is the University of California Los Angeles (UCLA) Physical Activity Score at 12 months. Secondary outcomes include: physical function, disability, health-related quality of life, kinesiophobia, perceived pain, self-efficacy and health resource utilisation.

ETHICS AND DISSEMINATION: Research ethics committee (REC) approval was granted by the NRES Committee South Central (Oxford B - 18/SC/0423). Dissemination of results will be through peer-reviewed, scientific journals and conference presentations.

TRIAL ISRCTN REGISTRATION NUMBER: ISRCTN29770908

PROTOCOL VERSION: Version 4.0 – Date: 17th September 2019

STATUS: trial recruitment is ongoing and is expected to be completed by 1st April 2020.

Keywords: arthroplasty; osteoarthritis; rehabilitation; physical activity; exercise; cognitive behavioural

STRENGTHS AND LIMITATIONS OF THIS STUDY

- The effectiveness of a behaviour change physiotherapy intervention to increase physical activity compared to usual rehabilitation after THR or TKR will be demonstrated with a pragmatic clinical trial design.
- Functional, behavioural and psychological outcomes will provide evidence to determine the mechanisms by-which the intervention is or is not effective.
- A multi-centre recruitment approach will provide greater external validity across population characteristics in England.

For peer review only - http://bmjopen.bmj.com/site/about/guidelines.xhtml

BACKGROUND

Total Hip (THR) and Total Knee Replacement (TKR) are two highly successful orthopaedic procedures which reduce pain for people with osteoarthritis.[1,2] Over 206,000 THR and TKRs were performed in the United Kingdom (UK) in 2018.[1] Approximately 90% of patients are satisfied following THR and TKR,[2] with significant improvements in pain and physical function after three to 12 months.[2,3]

Historically, it has been assumed that people become more active following THR or TKR through the amelioration of joint pain.[4] However, the current literature suggests physical activity, at best, remains the same from pre- to post-operatively, and in some instances declines.[4,5]

People following THR and TKR have reported a number of challenges which make engaging in physical activity difficult, most notably psychosocial barriers and fear avoidance beliefs.[6] Such barriers include receiving insufficient and inconsistent information on being more physically active, fear of damaging joint replacements and causing pain, and not being able to goal-set or problem-solve physical activities within individual's lifestyles.[6] Whilst previous international guidance as knowledged the importance of physical activity on health and wellbeing, people following THR and TKR have acknowledged these difficulties in being more active.[6] They have cited limited support or guidance currently offered on how to overcome these problems post-operatively.[6]

Not being physically active after joint replacement can have a major negative impact on a person's health and a burden on the National Health Service (NHS). Medical co-morbidities are common in this population. These include hypertension (56%),[7] cardiovascular disease (20%),[8] diabetes (16%)[8] and multi-joint pain (57%).[7] Twenty-seven percent of people who undergo joint replacement have three to four comorbidities.[8] Medical comorbidities have a significant negative impact on both health-related quality of life (HRQoL) and result in a societal burden.[9,10] Participating in regular physical activity can decrease the risk of cardiovascular disease by 52%,[11] diabetes by 65%,[12] and some cancers by 40%.[13] It is associated with a reduction in all-cause mortality by 33% and cardiovascular mortality by 35%.[14]

Current rehabilitation following THR and TKR in the UK, as advocated by the British Orthopaedic Association, centres around regaining joint movement, strength and gait (walking pattern) re-education.[15] There is currently no evidence informing patients or healthcare professionals on how to increase physical activity following THR and TKR. Previous research has demonstrated that behaviour-change interventions can effectively increase physical activity across the lifespan.[16-18] However, following joint replacement, people have specific psychological needs and challenges which differ to the non-joint replacement population.[6] Therefore, a specific intervention tailored to this population's health beliefs, including fear avoidance regarding implant survival, dislocation and increased knowledge on the impact of physical inactivity on other comorbidities, is required. Accordingly, the purpose of this trial is to answer the research question "following a primary THR or TKR, does a group exercise and behaviour-change intervention targeted to increase physical activity participation increase HRQoL and clinical outcomes over the initial 12 post-operative month compared to group exercise alone?"

METHODS AND DESIGN

For peer review only - http://bmjopen.bmj.com/site/about/guidelines.xhtml

Trial Design

This is a two-arm, pragmatic, parallel, multi-centre, randomised controlled superiority trial (RCT) to assess the effectiveness of a group exercise and behaviour-change intervention aimed to increase physical activity in people following THR or TKR. Nine UK NHS hospitals are involved in the trial across England. The study flow chart is presented as **Figure 1**. **Table 1** presents a summary of trial objectives, outcome measures and time points.

Participants

A minimum of 260 participants will be recruited.

Participants are eligible if they are:

- Due to undergo primary (first-time) unilateral THR or TKR where the indication for surgery is degenerative joint pathology (not trauma).
- Aged 18 years and over.
- Classified as 'moderately inactive' or 'inactive' using on the General Practice Physical Activity Questionnaire (GPPAQ).[19]
- Have a Charlson Comorbidity Index (CCI) of ≥ 1 point.[20,21]

Participants are ineligible if they have:

- An absolute contraindication to exercise such as severe cardiovascular or pulmonary disease.
- Cognitive impairment defined as an Abbreviated Mental Test Score (AMTS)[22] of <8.
- A usual place of residence that is a care home.
- Already enrolled onto another trial investigating physical activity, exercise adherence or behavioural therapy interventions.
- An inability to read and/or comprehend English.
- No access to a working telephone.

Recruitment

Potential participants will be identified from UK NHS hospital trusts by the clinical team once they have been listed for THR or TKR. They will be asked whether they would like to know more about the PEP-TALK trial. There are two options for this: either they will be given a copy of the Participant Information Sheet (PIS) and asked to contact a member of the Clinical Research Network (CRN) research team who will provide more information or will be asked whether they are happy for a member of the CRN research team to contact them directly with more information about the trial. Verbal consent for the approach will be documented by a clinical team member in the medical notes.

Potential participants will be asked to read the PIS and asked to discuss their potential participation with anyone who they feel would provide useful advice such as friends, family member or carers. The number of people provided with the PIS will be recorded to monitor how many participants are assessed for initial eligibility and sent the PIS.

Eligible patients who agree to participate will provide their consent during the pre-operative assessment appointment. Participant's eligibility will then be verified by reviewing the medical notes and by interviewing the participant using the screening log, Charlson Comorbidity Index (CCI) and General Practice Physical Activity Questionnaire (GPPAQ) tools.

For peer review only - http://bmjopen.bmj.com/site/about/guidelines.xhtml

Written informed consent will be obtained prior to any trial-specific procedures being performed. The baseline case report form (CRF) will be completed at the pre-operative assessments once consent has been taken. After the TKR or THR, eligibility will be confirmed by the site research team member reviewing the post-operative notes.

Randomisation, Blinding and Allocation Concealment

Consented participants will be randomised (1:1) to the group exercise and behaviour-change intervention (intervention group) or group exercise alone intervention (control group) using the centralised computer randomisation service RRAMP (https://rramp.octru.ox.ac.uk) provided by the Oxford Clinical Trials Research Unit (OCTRU). This will either be undertaken directly by the site's research facilitator or by contacting the trial office over the telephone, who will access the system on their behalf. Randomisation will be undertaken using a minimisation algorithm to ensure balanced allocation of participants across the two treatment groups, stratified by:

- Hospital site
- Type of joint replacement (THR or TKR)
- CCI of 1-3 versus ≥4[20,21]

The minimisation algorithm will be seeded using simple randomisation and will have a probabilistic element introduced to ensure unpredictability of treatment assignment.

Baseline data will be collected during the pre-operative assessment appointment and prior to randomisation. Therefore, the central randomisation system will issue a screening identifier (ID). Once the participant has been randomised, the central randomisation system will provide a participant ID to be used on all subsequent data collection forms. Sites will be responsible for linking the screening ID to the participant ID, with all linking information remaining at sites. This will be double-checked across the CRFs to ensure that the correct paperwork has been provided to the correct participant.

Due to the nature of the intervention, participants and those delivering the rehabilitation will be aware of the treatment allocation. By virtue of this design, it is not possible to blind participants, physiotherapists or the site researchers.

Change to the randomisation allocation: An amendment was made to the randomisation ratio in the minimisation algorithm from 1:1 to 2:1 (Experimental Intervention: Usual Care) after 75 participants had been randomised. The expected allocation ratio at the end of trial is likely to approximately 1.5:1. This was done to ensure a greater number of people are allocated to the experimental intervention, which is a group-based intervention. As the experimental intervention was designed to have three or more people per group, early sites have found it difficult to consistently reach this level of participant numbers with the original 1:1 randomisation allocation. This change was made to reduce the risk of small numbers in the experimental group. The Trial Management Group (TMG) agreed this on 15th August 2019. The sample size was increased to 260 to account for this change. This was approved by the sponsor, Data and Safety Monitoring Committee (DSMC) and research ethics committee.

Intervention

Usual Care

This will be received by both control and intervention groups.

For peer review only - http://bmjopen.bmj.com/site/about/guidelines.xhtml

In the in-patient setting, all participants will be seen by a physiotherapist a minimum of daily on weekdays, and will follow their hospital's standard post-operative pathway. Average hospital length of stay for people after THR and TKR is five days.[1] Rehabilitation consists of gait re-education, exercises and advice regarding transferring from bed to chair, toileting and dressing. Advice on continuation of gait progression and activities of daily living are encouraged by the physiotherapy team. Once considered medically fit and safe by the multidisciplinary team, patients are discharged. These follow current, routine, nationwide practice.[23]

All participants attend six-weekly, 30-minute group-based exercise classes within each hospital trust's physiotherapy department after THR or TKR. These groups consist of up to 12 people and commence within four weeks post-randomisation. The principles regarding prescription of group exercises to increase range of motion, strength and gait pattern are consistent. Whilst the rehabilitation of THR and TKR focuses on overall lower limb function, all participants following a THR focus on hip exercises, whereas those following a TKR focus on knee exercises. For this trial, exercise diaries will be provided and maintained by participants to monitor exercise performance and compliance within and outside the exercise groups for the six-week intervention period.

Experimental intervention

Participants randomised to the experimental group will receive the same in-hospital care and physiotherapy prior to hospital discharge. They will also receive the same six-weekly, group-based 30-minute exercise session. The only difference between the two groups is the addition of a 30-minute group-based (up to 12 people) behaviour change approach intervention prior to the routine 30 minutes of exercise and three telephone-follow-up calls two, four and six weeks after the last group-based session (the PEP-TALK intervention).

The PEP-TALK intervention is theoretically-based within the cognitive-behavioural model of understanding. It uses evidence-based behaviour change techniques to target internal (cognitions and behaviours regarding physical activity) and external factors (social and environmental barriers). It specifically aims to target self-efficacy beliefs and fear-avoidant behaviours. A senior health psychologist (BF), physiotherapist/cognitive-behavioural therapist (ZH) and senior clinical academic physiotherapist (TS) developed an in-person, one-day training session. The training was delivered by BF and TS to all physiotherapists in the trial who were delegated to deliver the PEP-TALK intervention. The training included role-play, knowledge and understanding testing and supplementary materials to support on-going learning. All physiotherapists were invited to contact the training team (BF, ZH,TS) to clarify any training queries throughout the trial.

In the PEP-TALK sessions, through group discussion, participants and physiotherapists will be encouraged to develop a positive therapeutic alliance, where the physiotherapist will generate an environment of trust and belief around individual challenges participant's have, to support them to overcome these for sustained physical activity adoption. A treatment log will be completed by the physiotherapists to record the components of what is discussed across the group in each session.

The PEP-TALK intervention also includes a home-practice element. Participants will be supported with skills developed in the group, to work at home on challenges, barriers and facilitators to physical activity behaviour. The 'home-work' workbook aims to translate and develop the skills learnt within the group session into daily life activities. The workbook is designed to be used during the six sessions although participants are encouraged to keep this as a record and prompt for long-term behaviour change. The workbook will be referred to in the telephone follow-up calls. The workbook encourages

For peer review only - http://bmjopen.bmj.com/site/about/guidelines.xhtml

reflective activities such as recording physical, emotional and cognitive barriers and facilitators to physical activity. It also offers problem-solving techniques such as “can you break down a large physical activity (e.g. ‘getting the house all cleaned for the family coming over’) into tasks which you can ‘prioritise’, ‘plan’, ‘tolerance level’ and ‘evaluate.’” The workbook also includes other activities to encourage pacing and behaviour modification, goal-setting to the individual’s health and social needs and techniques to challenge fear-avoidant behaviours. Education on exercise and the detrimental effects of physical inactivity will also be discussed. Participants will complete this with their home exercise plan which solely focuses on lower limb exercises rather than their behaviours and thoughts around physical activity.

The same trained physiotherapists who deliver the PEP-TALK intervention will, once the participant has completed their group sessions, deliver three, 20-minute telephone follow-up calls. These will occur two, four and six weeks after completing the group sessions. During these follow-up telephone calls, participant’s goals will be reviewed, any barriers to the completion of these goals will be identified, and the physiotherapist will review any ‘unhelpful’ and ‘helpful’ thoughts or feelings towards physical activity which may have arisen since the last consultation. Each telephone call will close with the development of longer-term physical activity goal-setting and promotion of empowerment towards physical activity participation using the behavioural principles instilled during the group intervention. A log will be collected by the physiotherapist to record the components of what is discussed during the telephone calls.

Delivery

Both the PEP-TALK intervention and exercise groups will be delivered as ‘rolling’ programmes. This means that new participants can join the group as it runs rather than waiting for a new ‘block’ of sessions to start. This will allow greater flexibility in allocating participants to join group sessions, whilst also avoiding a delay between post-operative referral and starting the sessions. The flexible nature of the experimental intervention also makes this feasible, with no ordering of the content of intervention sessions which would preclude such a rolling programme.

Contamination

The physiotherapists who deliver the behaviour change sessions will be taught the skills required to deliver the experimental intervention. These physiotherapists will not be permitted to deliver the control exercise group intervention during the trial period (and vice versa). This approach mitigates the risk of contamination. Due to the interventions being delivered in an out-patient setting, there is a reduced risk of participants sharing their knowledge and experience of the interventions between the control and intervention groups, further minimising the risk of between-group contamination. Participants in the control group will not be permitted to join the experimental intervention group’s exercise sessions (and vice versa). This will also reduce the risk of between-group contamination.

Co-Interventions

During the course of follow-up, participants may require further interventions as part of their recovery following surgery as per routine NHS practice. Further clinical interventions will be permitted for trial participants without the participant having to withdraw. If a participant receives additional treatment to the trial intervention, the details of the treatment received and the reasons will be collected.

Quality Assessment

The trial will be monitored and audited in accordance with the current approved protocol, good clinical practice,[24] relevant regulations and standard operating procedures (SOPs).

For peer review only - http://bmjopen.bmj.com/site/about/guidelines.xhtml

All designated physiotherapists who deliver the usual care group exercises will be taught about the standardised control intervention procedures, i.e. clarification on use of exercise diaries and treatment logs which are additional to usual care.

Designated physiotherapists delivering the behaviour change intervention will attend a one-day, face-to-face course where they will be taught the intervention and processes involved by a member of the PEP-TALK team who developed the intervention (BF, ZH, TS). In addition, to assess the fidelity to the trial intervention, a health psychologist (BF) will undertake a debriefing telephone call with each physiotherapist after they have delivered their first group session. This will allow the trainer to re-enforce any learning required, address any uncertainties and to identify and correct any variation in the treatment protocol. Finally, each physiotherapist who delivers either the PEP-TALK or control group intervention will be monitored during a site visit at their 3rd or 4th intervention session. The PEP-TALK intervention physiotherapist will be monitored by a health psychologist (BF) or physiotherapist with expertise in health psychology (ZH). Control group interventions will be monitored by a practicing physiotherapist (TS). Sessions will be monitored against the protocol to determine whether there are issues around fidelity, contamination across groups or adherence/compliance of participants. Where further training is required, this will be instigated following these visits. Further monitoring visits will be co-ordinated as required.

Assessments

Baseline Assessment

Baseline data will be collected prior to randomisation during the pre-operative assessment appointment, once consent has been obtained. This will include: gender, age, measured height and weight, CCI, self-reported presence and location of multi-site joint pain, co-morbidities determined from the medical notes, AMTS, employment status and occupation (when appropriate). Participants will also complete measures on: physical activity (University of California Los Angeles (UCLA) Activity Scale;[25] physical function (Lower Extremity Functional Scale (LEFS);[26] disease-specific function (Oxford Hip Score [27] or Oxford Knee Score [28]); pain (numerical rating scale for pain); self-efficacy (Generalized Self-Efficacy Scale [29]); fear of movement or kinesiophobia (Tampa Scale for Kinesiophobia [30]); psychological distress (Hospital Anxiety and Depression Scale (HADS) [31]); HRQoL (EQ-5D-5L [32]) and the health utilisation questionnaire.

Follow-up data will be collected six- and 12-months post-randomisation. Questionnaires will be sent to participants by post from the trial office and returned using a pre-addressed, pre-paid envelope. If participants have not responded within 14 days of posting, the trial team will attempt to telephone the participant on up to two occasions to remind them to complete the questionnaires. If required, a second postage of the questionnaires will be provided if requested by the participant during these follow-up telephone calls. If participants wish not to complete the questionnaires, they will be provided with the opportunity to complete the UCLA Activity Scale and EQ-5D-5L questionnaires over the telephone. If these methods fail, the participant would be categorised as a non-responder for that time-point only.

In an effort to find evidence-based techniques to improve retention and recruitment to RCTs, a study within a trial (SWAT) will be undertaken. This will examine whether there is a difference in questionnaire response rate by printing the UCLA Activity Scale on pink rather than white coloured paper at the six-month time-point. Results of this SWAT will be reported separately to the ‘host’ trial.

For peer review only - http://bmjopen.bmj.com/site/about/guidelines.xhtml

Outcome Measures

The data collection schedule is presented in **Table 2.**

Primary Outcome

- UCLA Activity Scale.[25] This is a reliable and valid self-reported tool to assess physical activity.[33,34] It assesses global activity levels with a grading system of one out of 10 points where one equates to "wholly inactive, dependent on others, and cannot leave residence" and 10 refers to "regularly participants in impact sports".[25]

Secondary Outcomes

- LEFS.[26] This is a valid and reliable measure of functional impairment in patients with lower extremity muscloskeletal conditions (Mehta et al, 2016).

- Oxford Hip Score (OHS) [27] or Oxford Knee Score (OKS).[28] Both measures have demonstrated good validity and reliability for people undergoing THR or TKR.[35]

- Numerical Rating Scale (NRS) for Pain. The NRS has been previously reported as reliable and valid to assess pain.[36]

- Generalized Self-Efficacy Scale.[29] This is a reliable and valid measure of self-efficacy.[37,38].

- The Tampa Scale for Kinesiophobia.[30] This is a valid and reliable measure of kinesiophobia.[39,40]

- HADS.[31] This has been shown as a reliable and valid measure of anxiety and depression.[41]

- Complications or adverse events (self-reported) which may include: wound or joint infection, joint dislocation, delayed hospital discharge (measured by length of stay), falls or musculoskeletal injuries and exacerbations of multi-site joint pain. Adverse events and serious adverse events will be reported as per the clinical trial unit's SOP.

- Health economic and health resource utilisation. We will collect HRQoL using the EQ-5D-5L.[33] Primary sources (i.e. participant log books) will be used to record the duration of the assessment visits, number and duration of scheduled group sessions, equipment, consumables, educational material, behaviour change intervention training time; number and duration of scheduled telephone calls. Frequency of health care resource use will be collected through patient self-reported questionnaires at follow-ups. The categories of NHS resource use that will be collected include treatment costs, outpatient visits, any related-inpatient admissions, rehabilitation visits and any treatment for recurrences and adverse events. Non-medical costs such as caregiving and productivity loss for those in employment at each follow-up will be collected.

Data Analysis

For peer review only - http://bmjopen.bmj.com/site/about/guidelines.xhtml

Sample Size

Originally, 250 participants (125 per arm) are required to detect a standardised effect size of 0.4 with 80% power and 5% (two-sided) significance, and allowing for 20% loss to follow-up. These calculations are based on the primary outcome, UCLA Activity Scale, at 12 months, assuming a baseline standard deviation of 2.5 and a between-group difference of one.[33] The minimally clinically important difference (MCID) has been reported as a within person difference of 0.92 points.[33]

The sample size was increased to 260 to account for the amendment in randomisation proceeedure from 1:1 to 2:1 group allocation, as described earlier.

Statistical Analysis

A detailed statistical analysis plan (SAP) will be drafted early in the trial and will be finalised prior to any primary outcome analysis. This will be reviewed and will receive input from the Trial Steering Committee (TSC) and the DSMC.

All analyses will be undertaken on the intent-to-treat population, i.e. patients will be analysed as they were randomised regardless of the treatment received. Sensitivity analyses will be undertaken on the per-protocol population to assess a range of potential biases that could have resulted from loss-to-follow-up, protocol deviations and withdrawal (including mortality). Numerical and graphical summaries of all data will be presented including descriptions of missing data at each level.

Estimates of treatment effects will be reported with 95% confidence intervals. The primary outcome measure, UCLA Activity Scale at 12 months post-randomisation, will be analysed using a mixed-effects linear regression model adjusted for baseline activity, six month time point and the stratification factors. Centre will be included as a random-effect to take account of their potential heterogeneity and type of operation, comorbidity index, and other time points (baseline and six months) will be included as fixed-effects. Treatment by six month time point interaction will also be included in the model to allow time specific treatment effects to be calculated The adjusted difference in the means with corresponding 95% confidence interval will be reported together with the mean and 95% confidence intervals for each treatment group. If not normally distributed, then transformation to normality will be considered. If this is not possible, non-parametric techniques (for example Mann-Whitney or Kruskal-Wallis Test) will be used with no adjustment. Consistency of any observed intervention effect will be explored using forest plots for various subgroups including site, type of operation (TKR and THR) and comorbidity index using interactions. No formal testing of interaction effects will be undertaken as the trial is not powered to detect these and the effect of the intervention is expected to be similar in the THR and TKR subgroups, although their baseline physical activity may be different, which will be taken into account in the analysis.

Similar methods will be used to analyse continuous secondary outcomes. Binary and categorical secondary outcomes, such as complications, will be tabulated to show frequencies and percentages in each arm. Chi-squared tests or logistic regression will be used to assess statistical significance.

Missing data will be minimised by careful data management. Missing data will be described with reasons given where available. The number and percentage of individuals in the missing category will be presented by treatment arm.

The nature and mechanism for missing variables and outcomes will be investigated, and if appropriate, multiple imputation will be used. Sensitivity analyses will be undertaken assessing the underlying

For peer review only - http://bmjopen.bmj.com/site/about/guidelines.xhtml

missing data assumptions. Any imputation techniques will be fully described in the SAP. Subgroup analyses, complier average causal effect (CACE) analysis and mediation analyses will be undertaken after being fully specified in the SAP. Compliance with the intervention is defined as attending at least four of six group sessions with a minimum of three participants in the group for these sessions and receiving at least two of the three follow-up phone calls. A priori mediation analysis moderators will include self-efficacy, fear avoidance, psychological distress to compare the mediation pathways presented in the BeST intervention [42] to the PEP-TALK intervention.

All data collected on data collection forms will be used, since only essential data items will be collected. No data will be considered spurious in the analysis since all data will be checked and cleaned before analysis.

<u>Health Economics</u>

Respecting an *efficient trial design*, only if the data indicates clinical effectiveness of the experimental intervention (for the UCLA Activity Scale and LEFS), will additional funding be sought to analyse the health economic and utilisation data, to determine the cost-effectiveness of the intervention.

<u>Data Management</u>

All data will be processed according to the Data Protection Act 2018.[43] All documents will be stored safely in confidential conditions. Trial-specific documents, except for the signed consent form and follow-up contact details, will refer to the participant with a unique study participant number, not by name. Participant identifiable data will be stored separately from trial data. All trial data will be stored securely in offices or online in secure trial databases, only accessible by the central trial team in Oxford and authorised personnel.

Trial Status

The trial is funded for 36 months and commenced in August 2018. Recruitment is expected to be complete by April 2020 with the final follow-up visit for the final participant completed by April 2021. The trial will be completed by August 2021.

Site Locations

Orthopaedic services providing THR and TKRs in nine hospital trusts (eight recruiting sites) and health providers: (Norfolk and Norwich University Hospitals NHS Foundation Trust, Lewisham and Greenwich University Hospitals NHS Foundation Trust, Oxford University Hospital NHS Foundation Trust, City Hospitals Sunderland NHS Foundation Trust, Norwich Spire, Barts Health NHS Trust, North Middlesex University Hospital NHS Trust, St George's University Hospital NHS Foundation Trust (treatment site only), Epsom and St Helier University Hospitals NHS Foundation Trust (recruitment site only)).

For peer review only - http://bmjopen.bmj.com/site/about/guidelines.xhtml

Patient and Public Involvement

Patient involvement began during protocol development and continues throughout the trial. A patient-member will attend all TSC meetings. The same patient-member is a co-investigator, providing insights into the trial conduct, particularly on data collection processes, and will help interpret the findings to inform on the implications of the research during the trial's dissemination phase.

Ethics and Dissemination

Ethical approval was gained from the South Central (Oxford B) Research Ethics Committee (Approval Date: 23Oct2018; Reference Number: 18/SC/0423). The trial was prospectively registered (ISRCTN29770908). A DSMC and TSC was appointed to independently review the data on safety, protocol adherence and recruitment to the trial. Direct access will be granted to authorised representatives from the sponsor and host institution for monitoring and/or audit of the trial to ensure compliance with regulations. Annoymised data will be shared outside the research team when required. Researchers outside the trial team may formally request for a specific data set using a data request form, which will be part of the Data Management Plan (DMP). All such requests will need to be approved by the TMG.

Reporting of the trial will be consistent with the CONSORT 2010 Statement (patient reported outcomes and non-pharmcological interventions)[44] and Template for Intervention Description and Replication (TIDieR)[45] guidelines. A summary of the results and trial materials will be made available via the trial website on completion of the trial. We will submit the final report to a peer-reviewed academic journal.

DISCUSSION

This paper presents the research protocol for the PEP-TALK trial. Following a THR and TKR, only 50% of people reach World Health Organization recommended levels of physical activity.[15,46,47] Those who are least likely to meet these levels are people with a higher body mass index and with comorbidities.[48] These patients have the most to gain from being more physically active. If this trial's experimental intervention is shown to be effective, this intervention could have a significant and sustained impact on improving the management of comorbidities such as diabetes, cardiovascular diseases, depression and hypertension for over 103,000 people annually in England and Wales.[1] It is proposed that, in such an instance, this should be considered for implementation in healthcare services. This could help address the global challenge which multi-morbidities and an ageing population are expected to have on health and social care services.

DECLARATIONS AND ACKNOWLEDGEMENTS

Contributors: TS, BF, SJD, SL, AO, CH, VB, SP researched the topic and devised the study. TS, SP, AO, BF, SJD, CH, VB, MEP, SL provided the first draft of the manuscript. AO, SD provided statistical oversight. TS, SP, BF, AO, SJD, CH, VB, MEP, SL contributed equally to manuscript preparation. TS acts a guarantor.

For peer review only - http://bmjopen.bmj.com/site/about/guidelines.xhtml

Funding: The research is supported by the National Institute for Health Research (NIHR) Research for Patient Benefit grant (PB-PG-1216-20008). The views expressed are those of the author(s) and not necessarily those of the NHS, the NIHR or the Department of Health and Social Care.

Competing interests: None declared.

Disclaimer: TS is supported by the National Institute for Health Research (NIHR) Oxford Biomedical Research Centre (BRC). The views expressed are those of the author(s) and not necessarily those of the NHS, the NIHR or the Department of Health and Social Care.

Ethics approval: Ethical approval was gained from the South Central (Oxford B) Research Ethics Committee (Approval Date: 23Oct2018; Reference Number: 18/SC/0423).

Trial Sponsor: University of Oxford (Clinical Trials and Research Governance Team. Joint Research Office, 1st floor, Boundary Brook House, Churchill Drive, Headington, Oxford OX3 7GB. Email: ctrg@admin.ox.ac.uk. The views expressed are those of the author(s) and not necessarily those of the sponsor.

Provenance and peer review: Not commissioned; externally peer reviewed.

PEP-TALK Collaborators: Mr Steve Algar (Banbury, PPI Representative), Dr Zara Hansen (ZH) (University of Oxford), Professor Karen Barker (Principal Investigator - Oxford University Hospital NHS Foundation Trust (OUH)**,** Mr Ian Smith (Principal Investigator **-** Lewisham and Greenwich University Hospitals NHS Foundation Trust), Professor Iain McNamara (Principal Investigator (Norfolk and Norwich University Hospitals NHS Foundation Trust (NNUH) & Spire Norwich), Mr Michael Dunn (Principal Investigator - St George's University Hospital NHS Foundation Trust), Mrs Dawn Lockey (Principal Investigator - City Hospitals Sunderland NHS Foundation Trust), Mr Sonny Driver (Principal Investigator – North Middlesex University Hospital NHS Trust), Mrs Jamila Kassam (Barts Health NHS Trust), Mr Peter Penny (Norwich Spire, Norwich), Mrs Celia Woodhouse, Mrs Tracey Potter and Mrs Helena Daniell (NNUH), Mr Alex Herring and Mrs Yan Cunningham (City Hospitals Sunderland NHS Foundation Trust), Irrum Afzal (South West London Elective Orthopaedic Centre), Mr Maninderpal Matharu (Barts Health NHS Trust) and Mrs Tamsin Hughes, Ms Erin Hannink and Mrs Michelle Moynihan (OUH).

Oversight Committee Membership: TSC Members: Professor David Deehan (Newcastle University), Dr Emma Godfrey (Kings College London), Dr Neil Artz (University of the West of England), Mr Steve Algar (PPI representative). **DSMC Members:** Dr Lindsey Smith (University of the West of England & Weston Area Health NHS Trust), Dr Dipesh Mistry (University of Warwick), Mr Paul Baker (South Tees Hospital NHS Foundation Trust)

Provenance and peer review: Not commissioned; externally peer reviewed.

Patient consent for publication: Not required.

Word count: 4360

FIGURE AND TABLE LEGENDS

Figure 1: Study flow chart

For peer review only - http://bmjopen.bmj.com/site/about/guidelines.xhtml

Table 1: Data collection schedule

Table 2: PEP-TALK trial objectives, outcome measures and measurement time-points

REFERENCES

1. National Joint Registry. 12th Annual Report. Accessed 15.09.2016. Available at: http://www.njrcentre.org.uk/njrcentre/Portals/0/Documents/England/Reports/12th%20annual%20eport/NJR%20Online%20Annual%20Report%202015.pdf

2. Dailiana ZH, Papakostidou I, Varitimidis S, Liaropoulos L, Zintzaras E, Karachalios T, Michelinakis E, Malizos KN. Patient-reported quality of life after primary major joint arthroplasty: a prospective comparison of hip and knee arthroplasty. BMC Musculoskelet Disord. 2015 Nov 26;16:366.

3. Papakostidou I, Dailiana ZH, Papapolychroniou T, Liaropoulos L, Zintzaras E, Karachalios TS, Malizos KN. Factors affecting the quality of life after total knee arthroplasties: a prospective study. BMC Musculoskelet Disord. 2012 Jun 29;13:116.

4. Withers TM, Lister S, Sackley C, Clark A, Smith TO. Is there a difference in physical activity levels in patients before and up to one year after unilateral total hip replacement? A systematic review and meta-analysis. Clin Rehabil. 2016: In Press.

5. Smith TO, Hilton G, Mansfield M, Mann CJV, Sackley CM. Does physical activity change following hip and knee arthroplasty? An analysis of data from the Osteoarthritis Initiative. Physiotherapy. 2018; In Press.

6. Smith TO, Latham S, Maskrey V, Blyth A. Patients' perceptions of physical activity before and after joint replacement: a systematic review with meta-ethnographic analysis. Postgrad Med J. 2015 Sep;91(1079):483-91.

7. Inacio MC, Pratt NL, Roughead EE, Graves SE. Comparing co-morbidities in total joint arthroplasty patients using the RxRisk-V, Elixhauser, and Charlson Measures: a cross-sectional evaluation. BMC Musculoskelet Disord. 2015 Dec 10;16:385.

8. Peter WF, Dekker J, Tilbury C, Tordoir RL, Verdegaal SH, Onstenk R, Bénard MR, Vehmeijer SB, Fiocco M, Vermeulen HM, van der Linden-van der Zwaag HM, Nelissen RG, Vliet Vlieland TP. The association between comorbidities and pain, physical function and quality of life following hip and knee arthroplasty. Rheumatol Int. 2015 Jul;35(7):1233-41.

9. Smith E, Hoy DG, Cross M, Vos T, Naghavi M, Buchbinder R, Woolf AD, March L. PICO. The global burden of other musculoskeletal disorders: estimates from the Global Burden of Disease 2010 study. Ann Rheum Dis. 2014 Aug;73(8):1462-9.

10. Sampson UK, Fowkes FG, McDermott MM, Criqui MH, Aboyans V, Norman PE, Forouzanfar MH, Naghavi M, Song Y, Harrell FE Jr, Denenberg JO, Mensah GA, Ezzati M, Murray C. Global and

For peer review only

regional burden of death and disability from peripheral artery disease: 21 world regions, 1990 to 2010. Glob Heart. 2014 Mar;9(1):145-158.e21.

11. Lee CD, Folsom AR, Blair SN. Physical activity and stroke risk: a meta-analysis. Stroke. 2003 Oct;34(10):2475-81.

12. Laaksonen DE, Lindström J, Lakka TA, Eriksson JG, Niskanen L, Wikström K, Aunola S, Keinänen-Kiukaanniemi S, Laakso M, Valle TT, Ilanne-Parikka P, Louheranta A, Hämäläinen H, Rastas M, Salminen V, Cepaitis Z, Hakumäki M, Kaikkonen H, Härkönen P, Sundvall J, Tuomilehto J, Uusitupa M; Finnish diabetes prevention study. Physical activity in the prevention of type 2 diabetes: the Finnish diabetes prevention study. Diabetes. 2005 Jan;54(1):158-65.

13. Lee IM. Physical activity and cancer prevention--data from epidemiologic studies. Med Sci Sports Exerc. 2003 Nov;35(11):1823-7.

14. Nocon M, Hiemann T, Müller-Riemenschneider F, Thalau F, Roll S, Willich SN. Association of physical activity with all-cause and cardiovascular mortality: a systematic review and meta-analysis. Eur J Cardiovasc Prev Rehabil. 2008 Jun;15(3):239-46.

15. Kersten RF, Stevens M, van Raay JJ, Bulstra SK, van den Akker-Scheek I. Habitual physical activity after total knee replacement. Phys Ther. 2012 Sep;92(9):1109-16.

16. French DP, Olander EK, Chisholm A, Mc Sharry J. Which behaviour change techniques are most effective at increasing older adults' self-efficacy and physical activity behaviour? A systematic review. Ann Behav Med. 2014 Oct;48(2):225-34.

17. Devereux-Fitzgerald A, Powell R, Dewhurst A, French DP. The acceptability of physical activity interventions to older adults: A systematic review and meta-synthesis. Soc Sci Med. 2016 Jun;158:14-23.

18. Friederichs SA, Oenema A, Bolman C, Lechner L. Long term effects of self-determination theory and motivational interviewing in a web-based physical activity intervention: randomized controlled trial. Int J Behav Nutr Phys Act. 2015 Aug 18;12:101.

19. Department of Health. 2013. General Practice Physical Activity Questionnaire. Accessed on 21.02.2017. Available at: https://www.gov.uk/government/publications/general-practice-physical-activity-questionnaire-gppaq

20. Charlson ME, Pompei P, Ales KL, MacKenzie CR. A new method of classifying prognostic comorbidity in longitudinal studies: development and validation. J Chronic Dis. 1987;40(5):373-83.

21. Charlson ME, Charlson RE, Briggs W, Hollenberg J. Can disease management target patients most likely to generate high costs? The impact of comorbidity. J Gen Intern Med. 2007; 22: 464-469.

22. Hodkinson HM. Evaluation of a mental test score for assessment of mental impairment in the elderly. Age Ageing 1972;1: 233–8.

23. Briggs TWR. 2012. Getting it right first time. Improving the quality of orthopaedic care within the National Health Service in England. Accessed 06.10.2016. Available at: http://www.gettingitrightfirsttime.com/downloads/briggsreporta4_fin.pdf

For peer review only - http://bmjopen.bmj.com/site/about/guidelines.xhtml

24. International Conference on Harmonisation Good Clinical Practice (ICH GCP). International Conference on Harmonisation Good Clinical Practice Recommendations. Accessed: 08 March 2018. Available at: https://www.ich.org/fileadmin/Public_Web_Site/ICH_Products/Guidelines/Efficacy/E6/E6_R1_Guideline.pdf

25. Zahiri CA, Schmalzried TP, Szuszczewicz ES, Amstutz HC. Assessing activity in joint replacement patients. J Arthroplasty. 1998;13:890–895

26. Binkley JM, Stratford OW, Lott SA, Riddle DL. The Lower Extremity Functional Scale (LEFS): Scale development, measurement properties, and clinical application. Physical Therapy 1999;79:371-383

27. Dawson J, Fitzpatrick R, Carr A, Murray D. Questionnaire on the perceptions of patients about total hip replacement. J Bone Joint Surg Br. 1996 Mar;78(2):185-90.

28. Dawson J, Fitzpatrick R, Murray D, Carr A. Questionnaire on the perceptions of patients about total knee replacement. J Bone Joint Surg Br. 1998 Jan;80(1):63-9.

29. Schwarzer R, Jerusalem M. Generalized Self-Efficacy scale. In J. Weinman, S. Wright, & M. Johnston, Measures in health psychology: A user's portfolio. Causal and control beliefs 1995, pp. 35-37.

30. Miller RP, Kori S, Todd D. The Tampa Scale: a measure of kinesiophobia. Clin J Pain 1991;7(1):51–52.

31. Zigmond AS, Snaith RP. The hospital anxiety and depression scale. Acta Psychiatrica Scandinavica. 1983; 67: 361–370.

32. EuroQol Group E (1990) EuroQol—a new facility for the measurement of health-related quality of life. Health Policy 16: 199–208.

33. SooHoo NF, Li Z, Chenok KE, Bozic KJ. Responsiveness of patient reported outcome measures in total joint arthroplasty patients. J Arthroplasty. 2015 Feb;30(2):176-91.

34. Naal FD, Impellizzeri FM, Leunig M. Which is the best activity rating scale for patients undergoing total joint arthroplasty? Clin Orthop Relat Res. 2009 Apr;467(4):958-65.

35. Harris K, Dawson J, Gibbons E, Lim CR, Beard DJ, Fitzpatrick R, Price AJ. Systematic review of measurement properties of patient-reported outcome measures used in patients undergoing hip and knee arthroplasty. Patient Relat Outcome Meas. 2016 Jul 25;7:101-8. doi: 10.2147/PROM.S97774.

36. Ferreira-Valente MA, Pais-Ribeiro JL, Jensen MP. Validity of four pain intensity rating scales. Pain. 2011 Oct;152(10):2399-404.

37. Bonsaksen T, Kottorp A, Gay C, Fagermoen MS, Lerdal A. Rasch analysis of the General Self-Efficacy Scale in a sample of persons with morbid obesity. Health Qual Life Outcomes. 2013 Nov 25;11:202. doi: 10.1186/1477-7525-11-202.

For peer review only

For peer review only - http://bmjopen.bmj.com/site/about/guidelines.xhtml

38. Barlow JH, Williams B, Wright C. The Generalized Self-Efficacy Scale in people with arthritis. Arthritis Care Res. 1996 Jun;9(3):189-96.

39. Lundberg MKE, Styf J, Carlsson SG. A psychometric evaluation of the Tampa Scale for Kinesiophobia – from a physiotherapeutic perspective. Physiotherapy Theory and Practice. 2004;20(2):121–133.

40. Bunketorp L, Carlsson J, Kowalski J, Stener-Victorin E. Evaluating the reliability of multi-item scales: a non-parametric approach to the ordered categorical structure of data collected with the Swedish version of the Tampa Scale for Kinesiophobia and the Self-Efficacy Scale. J Rehabil Med. 2005 Sep;37(5):330-4

41. Bjelland I, Dahl AA, Haug TT, Neckelmann D. The validity of the Hospital Anxiety and Depression Scale: an updated literature review. J Psychosom Res. 2002;52(2):69–77

42. Lamb SE, Lall R, Hansen Z, Castelnuovo E, Withers EJ, Nichols V, Griffiths F, Potter R, Szczepura A, Underwood M; BeST trial group. A multicentred randomised controlled trial of a primary care-based cognitive behavioural programme for low back pain. The Back Skills Training (BeST) trial. Health Technol Assess. 2010 Aug;14(41):1-253, iii-iv.

43. Data Protection Act. 2018. Accessed: 30 September 2019. Available at: http://www.legislation.gov.uk/ukpga/2018/12/contents/enacted

44. Schulz KF , Altman DG , Moher D , et al. CONSORT 2010 statement: updated guidelines for reporting parallel group randomised trials. BMC Med 2010;8:18

45. Hoffmann TC , Glasziou PP, Boutron I, et al. Better reporting of interventions: template for intervention description and replication (TIDieR) checklist and guide. BMJ 2014;348:g1687

46. Groen JW, Stevens M, Kersten RF, Reininga IH, van den Akker-Scheek I. After total knee arthroplasty, many people are not active enough to maintain their health and fitness: an observational study. J Physiother. 2012;58(2):113-6.

47. Wagenmakers R, Stevens M, Zijlstra W, Jacobs ML, van den Akker-Scheek I, Groothoff JW, Bulstra SK. Habitual physical activity behavior of patients after primary total hip arthroplasty. Phys Ther. 2008 Sep;88(9):1039-48.

48. Paxton EW, Torres A, Love RM, Barber TC, Sheth DS, Inacio MC. Total joint replacement: A multiple risk factor analysis of physical activity level 1-2 years postoperatively. Acta Orthop. 2016 Jul;87 Suppl 1:44-9.

For peer review only - http://bmjopen.bmj.com/site/about/guidelines.xhtml

1 **Table 1:** PEP-TALK trial objectives, outcome measures and measurement time-points

Objectives	Outcome Measures	Time-points
**Primary Objective**		
To compare physical activity participation at 12 months post randomisation between people who receive a group exercise and behaviour change intervention versus group exercise alone following THR or TKR.	University of California, Los Angeles (UCLA) activity scale	Baseline; 6 months; 12 months (primary time-point)
**Secondary Objectives**		
To compare the functional outcome between people who receive a group exercise and behaviour-change intervention versus group exercise alone following THR or TKR.	Lower Extremity Functional Scale (LEFS)	Baseline; 6 months; 12 months
To compare the disease-specific pain and function between people who receive a group exercise and behaviour-change intervention versus group exercise alone following THR or TKR.	Oxford Hip Score (OHS) [35] or Oxford Knee Score (OKS)	Baseline; 6 months; 12 months
To compare the perceived level of pain between people who receive a group exercise and behaviour-change intervention versus group exercise alone following THR or TKR.	Numerical Rating Scale (NRS) for Pain	Baseline; 6 months; 12 months
To compare participant's self-efficacy between those who receive a group exercise and behaviour-change intervention versus group exercise alone following THR or TKR.	Generalized Self-Efficacy Scale (GSES)	Baseline; 6 months; 12 months
To compare participant's fear avoidance to movement between those who receive a group exercise and behaviour-change intervention versus group exercise alone following THR or TKR.	The Tampa Scale for Kinesiophobia	Baseline; 6 months; 12 months
To compare participant's psychological distress (anxiety and depression) between those who receive a group exercise and behaviour-change intervention versus group exercise alone following THR or TKR.	Hospital Anxiety and Depression Scale (HADS)	Baseline; 6 months; 12 months
To compare participant's complications and adverse events between those who receive a group exercise and behaviour-change intervention versus group exercise alone following THR or TKR.	Complications or adverse events recorded in the CRFs	6 months; 12 months
To collect cost-effectiveness data (health resource utilisation; direct and indirect costs) of a group exercise and a behaviour-change intervention versus group exercise alone following THR or TKR.	EQ-5D-5L; Bespoke health care resource self-reported questionnaire	6 months; 12 months

2

For peer review only - http://bmjopen.bmj.com/site/about/guidelines.xhtml

Table 2: Data collection schedule

Data	Time-points and Mode of Data Collection			
	Baseline	Intervention Period	Month 6 Post-Randomisation	Month 12 Post-Randomisation
	Face-to-Face	Face-to-Face	Postal	Postal
Age (years)	■			
Gender	■			
Weight (kg)	■			
Height (cm)	■			
Admission Date	■			
Operative procedure (THR or TKR)	■			
Side of Joint Replacement	■			
Duration of hip or knee symptoms	■			
Presence and location of multi-joint pain	■		■	■
ASA grade	■			
AMTS	■			
List of medical co-morbidities	■			
Physiotherapist exercise class log		■		
Patient in-session and home exercise diary		■		
Behaviour change intervention log (group and telephone) (physiotherapist completed)		■		
Charlson Comorbidity Score	■			
Employment status and current occupation (when appropriate)	■		■	■
UCLA Activity Score	■		■	■
Lower Extremity Functional Scale	■		■	■
Oxford Hip or Knee Score	■		■	■
Numerical Rating Scale – Pain	■		■	■
Generalized Self-Efficacy Scale	■		■	■
Tampa Scale for Kinesiophobia	■		■	■
Hospital Anxiety and Depression Scale	■		■	■
EQ-5D-5L	■		■	■
Health economic/Health utilisation questionnaire			■	■
Complications and adverse events			■	■

Shaded areas represent where these data are being collected.

Assessment intervals following randomisation; each follow-up interval +/- 1 month

AMTS – Abbreviated Mental Test Score; ASA – American Society of Anaesthesiologists classification; cm – centimetres; kg – kilograms; THR – total hip replacement; TKR – total knee replacement; UCLA – University College Los Angeles.

For peer review only

For peer review only - http://bmjopen.bmj.com/site/about/guidelines.xhtml

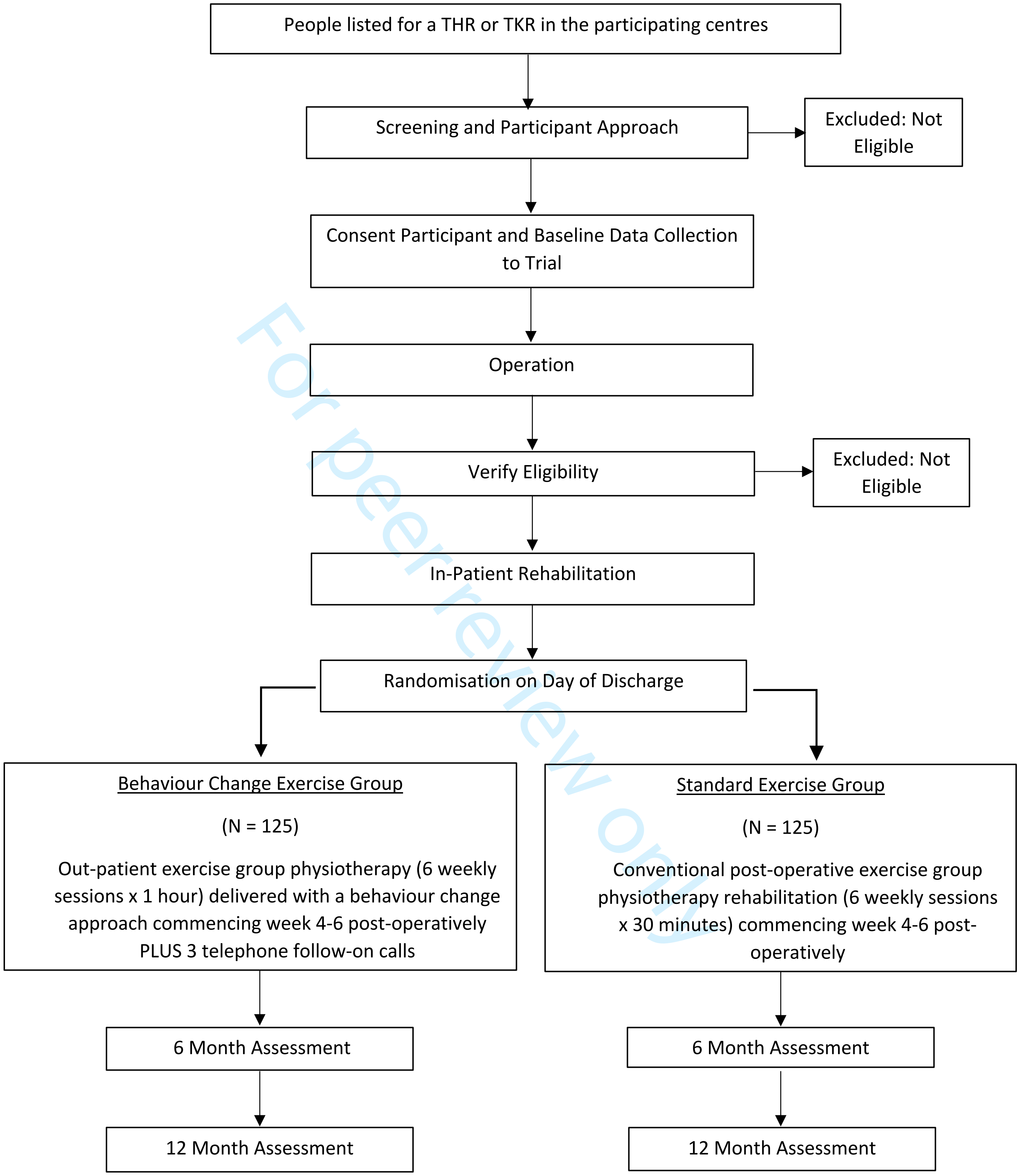

For peer review only - http://bmjopen.bmj.com/site/about/guidelines.xhtml

SPIRIT

STANDARD PROTOCOL ITEMS: RECOMMENDATIONS FOR INTERVENTIONAL TRIALS

SPIRIT 2013 Checklist: Recommended items to address in a clinical trial protocol and related documents*

Section/item	Item No	Description	Location in Text
**Administrative information**			
Title	1	Descriptive title identifying the study design, population, interventions, and, if applicable, trial acronym	Title
Trial registration	2a	Trial identifier and registry name. If not yet registered, name of intended registry	Abstract
	2b	All items from the World Health Organization Trial Registration Data Set	Abstract to ID
Protocol version	3	Date and version identifier	Abstract page
Funding	4	Sources and types of financial, material, and other support	Abstract page & Declarations
Roles and responsibilities	5a	Names, affiliations, and roles of protocol contributors	Title Page & Declarations
	5b	Name and contact information for the trial sponsor	Declarations
	5c	Role of study sponsor and funders, if any, in study design; collection, management, analysis, and interpretation of data; writing of the report; and the decision to submit the report for publication, including whether they will have ultimate authority over any of these activities	Declarations

For peer review only

	5d	Composition, roles, and responsibilities of the coordinating centre, steering committee, endpoint adjudication committee, data management team, and other individuals or groups overseeing the trial, if applicable (see Item 21a for data monitoring committee)	Data Management and Ethics and Dissemination
**Introduction**			
Background and rationale	6a	Description of research question and justification for undertaking the trial, including summary of relevant studies (published and unpublished) examining benefits and harms for each intervention	Background, Para 5
	6b	Explanation for choice of comparators	BACkground Para 3-4; Methods, Interventions
Objectives	7	Specific objectives or hypotheses	Background, Para 4
Trial design	8	Description of trial design including type of trial (eg, parallel group, crossover, factorial, single group), allocation ratio, and framework (eg, superiority, equivalence, noninferiority, exploratory)	Methods, Trial Design
**Methods: Participants, interventions, and outcomes**			
Study setting	9	Description of study settings (eg, community clinic, academic hospital) and list of countries where data will be collected. Reference to where list of study sites can be obtained	Methods, Site Locations & Declarations
Eligibility criteria	10	Inclusion and exclusion criteria for participants. If applicable, eligibility criteria for study centres and individuals who will perform the interventions (eg, surgeons, psychotherapists)	Methods, Participants

For peer review only

Interventions	11a	Interventions for each group with sufficient detail to allow replication, including how and when they will be administered	Methods, Interventions
	11b	Criteria for discontinuing or modifying allocated interventions for a given trial participant (eg, drug dose change in response to harms, participant request, or improving/worsening disease)	Methods, Contamination
	11c	Strategies to improve adherence to intervention protocols, and any procedures for monitoring adherence (eg, drug tablet return, laboratory tests)	Methods, Quality Assessment
	11d	Relevant concomitant care and interventions that are permitted or prohibited during the trial	Methods, Cointerventions
Outcomes	12	Primary, secondary, and other outcomes, including the specific measurement variable (eg, systolic blood pressure), analysis metric (eg, change from baseline, final value, time to event), method of aggregation (eg, median, proportion), and time point for each outcome. Explanation of the clinical relevance of chosen efficacy and harm outcomes is strongly recommended	Methods, Outcome Measures & Table 2
Participant timeline	13	Time schedule of enrolment, interventions (including any run-ins and washouts), assessments, and visits for participants. A schematic diagram is highly recommended (see Figure)	Figure 1
Sample size	14	Estimated number of participants needed to achieve study objectives and how it was determined, including clinical and statistical assumptions supporting any sample size calculations	Data Analysis, Sample Size
Recruitment	15	Strategies for achieving adequate participant enrolment to reach target sample size	Methods, Trial Status, Site Locations
**Methods: Assignment of interventions (for controlled trials)**			
Allocation:			

Sequence generation	16a	Method of generating the allocation sequence (eg, computer-generated random numbers), and list of any factors for stratification. To reduce predictability of a random sequence, details of any planned restriction (eg, blocking) should be provided in a separate document that is unavailable to those who enrol participants or assign interventions	Methods, Randomisation, Blinding and Allocation
Allocation concealment mechanism	16b	Mechanism of implementing the allocation sequence (eg, central telephone; sequentially numbered, opaque, sealed envelopes), describing any steps to conceal the sequence until interventions are assigned	Methods, Randomisation, Blinding and Allocation
Implementation	16c	Who will generate the allocation sequence, who will enrol participants, and who will assign participants to interventions	Methods, Randomisation, Blinding and Allocation
Blinding (masking)	17a	Who will be blinded after assignment to interventions (eg, trial participants, care providers, outcome assessors, data analysts), and how	Methods, Randomisation, Blinding and Allocation
	17b	If blinded, circumstances under which unblinding is permissible, and procedure for revealing a participant’s allocated intervention during the trial	Methods, Randomisation, Blinding and Allocation
**Methods: Data collection, management, and analysis**			
Data collection methods	18a	Plans for assessment and collection of outcome, baseline, and other trial data, including any related processes to promote data quality (eg, duplicate measurements, training of assessors) and a description of study instruments (eg, questionnaires, laboratory tests) along with their reliability and validity, if known. Reference to where data collection forms can be found, if not in the protocol	Methods, Outcome Measures & Table 2

	18b	Plans to promote participant retention and complete follow-up, including list of any outcome data to be collected for participants who discontinue or deviate from intervention protocols	Methods, Assessments
Data management	19	Plans for data entry, coding, security, and storage, including any related processes to promote data quality (eg, double data entry; range checks for data values). Reference to where details of data management procedures can be found, if not in the protocol	Methods, Assessment & Data Management
Statistical methods	20a	Statistical methods for analysing primary and secondary outcomes. Reference to where other details of the statistical analysis plan can be found, if not in the protocol	Data Analysis
	20b	Methods for any additional analyses (eg, subgroup and adjusted analyses)	Data Analysis
	20c	Definition of analysis population relating to protocol non-adherence (eg, as randomised analysis), and any statistical methods to handle missing data (eg, multiple imputation)	Data Analysis
**Methods: Monitoring**			
Data monitoring	21a	Composition of data monitoring committee (DMC); summary of its role and reporting structure; statement of whether it is independent from the sponsor and competing interests; and reference to where further details about its charter can be found, if not in the protocol. Alternatively, an explanation of why a DMC is not needed	Data Management & Ethics and Dissemination & Declarations
	21b	Description of any interim analyses and stopping guidelines, including who will have access to these interim results and make the final decision to terminate the trial	Data Management & Ethics and Dissemination
Harms	22	Plans for collecting, assessing, reporting, and managing solicited and spontaneously reported adverse events and other unintended effects of trial interventions or trial conduct	Data Management

For peer review only - http://bmjopen.bmj.com/site/about/guidelines.xhtml

Auditing	23	Frequency and procedures for auditing trial conduct, if any, and whether the process will be independent from investigators and the sponsor	Quality Assessment
**Ethics and dissemination**			
Research ethics approval	24	Plans for seeking research ethics committee/institutional review board (REC/IRB) approval	Declarations
Protocol amendments	25	Plans for communicating important protocol modifications (eg, changes to eligibility criteria, outcomes, analyses) to relevant parties (eg, investigators, REC/IRBs, trial participants, trial registries, journals, regulators)	Ethics and Dissemination
Consent or assent	26a	Who will obtain informed consent or assent from potential trial participants or authorised surrogates, and how (see Item 32)	Methods, Recruitment
	26b	Additional consent provisions for collection and use of participant data and biological specimens in ancillary studies, if applicable	Not applicable
Confidentiality	27	How personal information about potential and enrolled participants will be collected, shared, and maintained in order to protect confidentiality before, during, and after the trial	Data Management
Declaration of interests	28	Financial and other competing interests for principal investigators for the overall trial and each study site	Declarations
Access to data	29	Statement of who will have access to the final trial dataset, and disclosure of contractual agreements that limit such access for investigators	Data Management
Ancillary and post-trial care	30	Provisions, if any, for ancillary and post-trial care, and for compensation to those who suffer harm from trial participation	Not applicable
Dissemination policy	31a	Plans for investigators and sponsor to communicate trial results to participants, healthcare professionals, the public, and other relevant groups (eg, via publication, reporting in results databases, or other data sharing arrangements), including any publication restrictions	Ethics and Dissemination

For peer review only

	31b	Authorship eligibility guidelines and any intended use of professional writers	Not applicable
	31c	Plans, if any, for granting public access to the full protocol, participant-level dataset, and statistical code	Ethics and Dissemination
**Appendices**			
Informed consent materials	32	Model consent form and other related documentation given to participants and authorised surrogates	Available on request
Biological specimens	33	Plans for collection, laboratory evaluation, and storage of biological specimens for genetic or molecular analysis in the current trial and for future use in ancillary studies, if applicable	Not applicable.

*It is strongly recommended that this checklist be read in conjunction with the SPIRIT 2013 Explanation & Elaboration for important clarification on the items. Amendments to the protocol should be tracked and dated. The SPIRIT checklist is copyrighted by the SPIRIT Group under the Creative Commons “Attribution-NonCommercial-NoDerivs 3.0 Unported” license.

BMJ Open

Behaviour change physiotherapy intervention to increase physical activity following hip and knee replacement (PEP-TALK): study protocol for a pragmatic randomised controlled trial

Journal:	*BMJ Open*
Manuscript ID	bmjopen-2019-035014.R2
Article Type:	Protocol
Date Submitted by the Author:	08-May-2020

Complete List of Authors:	Smith, Toby O.; University of Oxford , Nuffield Department of Orthopaedics, Rheumatology and Musculoskeletal Sciences; University of East Anglia, Faculty of Medicine and Health Sciences<br>Parsons, Scott; University of Oxford, Nuffield Department of Orthopaedics, Rheumatology and Musculoskeletal Disorders<br>Fordham, Beth; University of Oxford , NDORMS<br>Ooms, Alexander; University of Oxford, Nuffield Department of Orthopaedics, Rheumatology and Musculoskeletal Disorders<br>Dutton, Susan; University of Oxford, CSM<br>Hing, Caroline; University of London St George's Molecular and Clinical Sciences Research Institute<br>Barber, Vicki; University of Oxford Nuffield Department of Orthopaedics Rheumatology and Musculoskeletal Sciences, Oxford Clinical Trials Unit<br>Png, May ee; University of Oxford, NDORMS<br>Lamb, Sarah; University of Oxford, Ndorms; University of Exeter, College of Medicine and Health Sciences

**Primary Subject Heading**:	Rheumatology
Secondary Subject Heading:	Surgery
Keywords:	Adult orthopaedics < ORTHOPAEDIC & TRAUMA SURGERY, Hip < ORTHOPAEDIC & TRAUMA SURGERY, Knee < ORTHOPAEDIC & TRAUMA SURGERY

SCHOLARONE™ Manuscripts

I, the Submitting Author has the right to grant and does grant on behalf of all authors of the Work (as defined in the below author licence), an exclusive licence and/or a non-exclusive licence for contributions from authors who are: i) UK Crown employees; ii) where BMJ has agreed a CC-BY licence shall apply, and/or iii) in accordance with the terms applicable for US Federal Government officers or employees acting as part of their official duties; on a worldwide, perpetual, irrevocable, royalty-free basis to BMJ Publishing Group Ltd ("BMJ") its licensees and where the relevant Journal is co-owned by BMJ to the co-owners of the Journal, to publish the Work in this journal and any other BMJ products and to exploit all rights, as set out in our licence.

The Submitting Author accepts and understands that any supply made under these terms is made by BMJ to the Submitting Author unless you are acting as an employee on behalf of your employer or a postgraduate student of an affiliated institution which is paying any applicable article publishing charge ("APC") for Open Access articles. Where the Submitting Author wishes to make the Work available on an Open Access basis (and intends to pay the relevant APC), the terms of reuse of such Open Access shall be governed by a Creative Commons licence – details of these licences and which Creative Commons licence will apply to this Work are set out in our licence referred to above.

Other than as permitted in any relevant BMJ Author's Self Archiving Policies, I confirm this Work has not been accepted for publication elsewhere, is not being considered for publication elsewhere and does not duplicate material already published. I confirm all authors consent to publication of this Work and authorise the granting of this licence.

For peer review only

BMJ OPEN

TITLE PAGE

Title: Behaviour change physiotherapy intervention to increase physical activity following hip and knee replacement (PEP-TALK): study protocol for a pragmatic randomised controlled trial

Authors: Smith TO,[1,2] Parsons SR,[1] Fordham B,[1] Ooms AG,[1,3] Dutton SJ,[1,3] Hing CB,[4] Barber VS,[1,3] Png ME,[1,3] Lamb SE[1,5] on behalf of the PEP-TALK Trial Collaborators.

Affiliations

1. Nuffield Department of Rheumatology, Orthopaedics and Musculoskeletal Sciences, University of Oxford, Botnar Research Centre, Windmill Road, Oxford, UK
2. Faculty of Medicine and Health Sciences, University of East Anglia, Norwich, UK
3. Oxford Clinical Trials Research Unit (OCTRU), University of Oxford, Windmill Road, Oxford, UK
4. Department of Trauma and Orthopaedics, St George's Hospital, St George's University Hospitals NHS Foundation Trust, Tooting, London, UK
5. College of Medicine and Health Sciences, University of Exeter, Exeter, UK

Corresponding Author: Dr Toby Smith, Nuffield Department of Rheumatology, Orthopaedics and Musculoskeletal Sciences, University of Oxford, Botnar Research Centre, Windmill Road, Oxford, UK. Email: toby.smith@ndorms.ox.ac.uk

Word Count: 4674

For peer review only

ABSTRACT

INTRODUCTION: Whilst total hip (THR) and knee replacements (TKR) successfully reduce pain associated with chronic joint pathology, this infrequently translates into increased physical activity. This is a challenge given that over 50% of individuals who undergo these operations are physically inactive and have medical comorbidities such as hypertension, heart disease, diabetes and depression. The impact of these diseases can be reduced with physical activity. This trial aims to investigate the effectiveness of a behaviour change physiotherapy intervention to increase physical activity compared to usual rehabilitation after THR or TKR.

METHODS AND ANALYSIS: The PEP-TALK trial is a multi-centre, open-labelled, pragmatic randomised controlled trial. 260 adults who are scheduled to undergo a primary unilateral THR or TKR and are moderately inactive or inactive, with comorbidities, will be recruited across eight sites in England. They will be randomised post-surgery, prior to hospital discharge, to either six, 30-minute weekly group-based exercise sessions(control), or the same six-weekly, group-based, exercise sessions each preceded by a 30-minute cognitive behaviour approach discussion group. Participants will be followed-up to 12 months by postal questionnaire. The primary outcome is the University of California Los Angeles (UCLA) Physical Activity Score at 12 months. Secondary outcomes include: physical function, disability, health-related quality of life, kinesiophobia, perceived pain, self-efficacy and health resource utilisation.

ETHICS AND DISSEMINATION: Research ethics committee (REC) approval was granted by the NRES Committee South Central (Oxford B - 18/SC/0423). Dissemination of results will be through peer-reviewed, scientific journals and conference presentations.

TRIAL ISRCTN REGISTRATION NUMBER: ISRCTN29770908

PROTOCOL VERSION: Version 4.0 – Date: 17th September 2019

STATUS: trial recruitment is ongoing and is expected to be completed by 1st April 2020.

Keywords: arthroplasty; osteoarthritis; rehabilitation; physical activity; exercise; cognitive behavioural

STRENGTHS AND LIMITATIONS OF THIS STUDY

- The effectiveness of a behaviour change physiotherapy intervention to increase physical activity compared to usual rehabilitation after THR or TKR will be demonstrated with a pragmatic clinical trial design.
- Functional, behavioural and psychological outcomes will provide evidence to determine the mechanisms by-which the intervention is or is not effective.
- A multi-centre recruitment approach will provide greater external validity across population characteristics in England.
- It is not possible to blind participants to the rehabilitation treatments given the participatory nature of the interventions.
- The group-based intervention may be challenging to ensure sufficient numbers within each group as participants enter the trial.

For peer review only - http://bmjopen.bmj.com/site/about/guidelines.xhtml

For peer review only

INTRODUCTION

Total Hip (THR) and Total Knee Replacement (TKR) are two highly successful orthopaedic procedures which reduce pain for people with osteoarthritis.[1,2] Over 206,000 THR and TKRs were performed in the United Kingdom (UK) in 2018.[1] Approximately 90% of patients are satisfied following THR and TKR,[2] with significant improvements in pain and physical function after three to 12 months.[2,3]

Historically, it has been assumed that people become more active following THR or TKR through the amelioration of joint pain.[4] However, the current literature suggests physical activity, at best, remains the same from pre- to post-operatively, and in some instances declines.[4,5]

People following THR and TKR have reported a number of challenges which make engaging in physical activity difficult, most notably psychosocial barriers and fear avoidance beliefs.[6] Such barriers include receiving insufficient and inconsistent information on being more physically active, fear of damaging joint replacements and causing pain, and not being able to goal-set or problem-solve physical activities within individual's lifestyles.[6] Whilst previous international guidance as knowledged the importance of physical activity on health and wellbeing, people following THR and TKR have acknowledged these difficulties in being more active.[6] They have cited limited support or guidance currently offered on how to overcome these problems post-operatively.[6]

Not being physically active after joint replacement can have a major negative impact on a person's health and a burden on the National Health Service (NHS). Medical co-morbidities are common in this population. These include hypertension (56%),[7] cardiovascular disease (20%),[8] diabetes (16%)[8] and multi-joint pain (57%).[7] Twenty-seven percent of people who undergo joint replacement have three to four comorbidities.[8] Medical comorbidities have a significant negative impact on both health-related quality of life (HRQoL) and result in a societal burden.[9,10] Participating in regular physical activity can decrease the risk of cardiovascular disease by 52%,[11] diabetes by 65%,[12] and some cancers by 40%.[13] It is associated with a reduction in all-cause mortality by 33% and cardiovascular mortality by 35%.[14]

Current rehabilitation following THR and TKR in the UK, as advocated by the British Orthopaedic Association, centres around regaining joint movement, strength and gait (walking pattern) re-education.[15] There is currently no evidence informing patients or healthcare professionals on how to increase physical activity following THR and TKR. Previous research has demonstrated that behaviour-change interventions can effectively increase physical activity across the lifespan.[16-18] However, following joint replacement, people have specific psychological needs and challenges which differ to the non-joint replacement population.[6] Therefore, a specific intervention tailored to this population's health beliefs, including fear avoidance regarding implant survival, dislocation and increased knowledge on the impact of physical inactivity on other comorbidities, is required. Accordingly, the purpose of this trial is to answer the research question "following a primary THR or TKR, does a group exercise and behaviour-change intervention targeted to increase physical activity participation increase HRQoL and clinical outcomes over the initial 12 post-operative month compared to group exercise alone?"

METHODS AND ANALYSIS

For peer review only - http://bmjopen.bmj.com/site/about/guidelines.xhtml

Trial Design

This is a two-arm, pragmatic, parallel, multi-centre, randomised controlled superiority trial (RCT) to assess the effectiveness of a group exercise and behaviour-change intervention aimed to increase physical activity in people following THR or TKR. Nine UK NHS hospitals are involved in the trial across England. The study flow chart is presented as **Figure 1**. **Table 1** presents a summary of trial objectives, outcome measures and time points.

Trial Participants

A minimum of 260 participants will be recruited.

Participants are eligible if they are:

- Due to undergo primary (first-time) unilateral THR or TKR where the indication for surgery is degenerative joint pathology (not trauma).
- Aged 18 years and over.
- Classified as 'moderately inactive' or 'inactive' using on the General Practice Physical Activity Questionnaire (GPPAQ).[19]
- Have a Charlson Comorbidity Index (CCI) of ≥ 1 point.[20,21]

Participants are ineligible if they have:

- An absolute contraindication to exercise such as severe cardiovascular or pulmonary disease.
- Cognitive impairment defined as an Abbreviated Mental Test Score (AMTS)[22] of <8.
- A usual place of residence that is a care home.
- Already enrolled onto another trial investigating physical activity, exercise adherence or behavioural therapy interventions.
- An inability to read and/or comprehend English.
- No access to a working telephone.

Recruitment

Potential participants will be identified from UK NHS hospital trusts by the clinical team once they have been listed for THR or TKR. They will be asked whether they would like to know more about the PEP-TALK trial. There are two options for this: either they will be given a copy of the Participant Information Sheet (PIS) and asked to contact a member of the Clinical Research Network (CRN) research team who will provide more information or will be asked whether they are happy for a member of the CRN research team to contact them directly with more information about the trial. Verbal consent for the approach will be documented by a clinical team member in the medical notes.

Potential participants will be asked to read the PIS and asked to discuss their potential participation with anyone who they feel would provide useful advice such as friends, family member or carers. The number of people provided with the PIS will be recorded to monitor how many participants are assessed for initial eligibility and sent the PIS.

Eligible patients who agree to participate will provide their consent during the pre-operative assessment appointment. Participant's eligibility will then be verified by reviewing the medical notes

For peer review only - http://bmjopen.bmj.com/site/about/guidelines.xhtml

and by interviewing the participant using the screening log, Charlson Comorbidity Index (CCI) and General Practice Physical Activity Questionnaire (GPPAQ) tools.

Written informed consent (**Supplementary File 1**) will be obtained prior to any trial-specific procedures being performed. The baseline case report form (CRF) will be completed at the pre-operative assessments once consent has been taken. After the TKR or THR, eligibility will be confirmed by the site research team member reviewing the post-operative notes.

Randomisation, Blinding and Allocation Concealment

Consented participants will be randomised (1:1) to the group exercise and behaviour-change intervention (intervention group) or group exercise alone intervention (control group) using the centralised computer randomisation service RRAMP (https://rramp.octru.ox.ac.uk) provided by the Oxford Clinical Trials Research Unit (OCTRU). This will either be undertaken directly by the site's research facilitator or by contacting the trial office over the telephone, who will access the system on their behalf. Randomisation will be undertaken using a minimisation algorithm to ensure balanced allocation of participants across the two treatment groups, stratified by:

- Hospital site
- Type of joint replacement (THR or TKR)
- CCI of 1-3 versus ≥4[20,21]

The minimisation algorithm will be seeded using simple randomisation and will have a probabilistic element introduced to ensure unpredictability of treatment assignment.

Baseline data will be collected during the pre-operative assessment appointment and prior to randomisation. Therefore, the central randomisation system will issue a screening identifier (ID). Once the participant has been randomised, the central randomisation system will provide a participant ID to be used on all subsequent data collection forms. Sites will be responsible for linking the screening ID to the participant ID, with all linking information remaining at sites. This will be double-checked across the CRFs to ensure that the correct paperwork has been provided to the correct participant.

Due to the nature of the intervention, participants and those delivering the rehabilitation will be aware of the treatment allocation. By virtue of this design, it is not possible to blind participants, physiotherapists or the site researchers.

Change to the randomisation allocation: An amendment was made to the randomisation ratio in the minimisation algorithm from 1:1 to 2:1 (Experimental Intervention: Usual Care) after 75 participants had been randomised. The expected allocation ratio at the end of trial is likely to approximately 1.5:1. This was done to ensure a greater number of people are allocated to the experimental intervention, which is a group-based intervention. As the experimental intervention was designed to have three or more people per group, early sites have found it difficult to consistently reach this level of participant numbers with the original 1:1 randomisation allocation. This change was made to reduce the risk of small numbers in the experimental group. The Trial Management Group (TMG) agreed this on 15th August 2019. The sample size was increased to 260 to account for this change. This was approved by the sponsor, Data and Safety Monitoring Committee (DSMC) and research ethics committee.

For peer review only - http://bmjopen.bmj.com/site/about/guidelines.xhtml

Intervention

Usual Care

This will be received by both control and intervention groups.

In the in-patient setting, all participants will be seen by a physiotherapist a minimum of daily on weekdays, and will follow their hospital's standard post-operative pathway. Average hospital length of stay for people after THR and TKR is five days.[1] Rehabilitation consists of gait re-education, exercises and advice regarding transferring from bed to chair, toileting and dressing. Advice on continuation of gait progression and activities of daily living are encouraged by the physiotherapy team. Once considered medically fit and safe by the multidisciplinary team, patients are discharged. These follow current, routine, nationwide practice.[23]

All participants attend six-weekly, 30-minute group-based exercise classes within each hospital trust's physiotherapy department after THR or TKR. These groups consist of up to 12 people and commence within four weeks post-randomisation. The principles regarding prescription of group exercises to increase range of motion, strength and gait pattern are consistent. Whilst the rehabilitation of THR and TKR focuses on overall lower limb function, all participants following a THR focus on hip exercises, whereas those following a TKR focus on knee exercises. For this trial, exercise diaries will be provided and maintained by participants to monitor exercise performance and compliance within and outside the exercise groups for the six-week intervention period.

Experimental intervention

Participants randomised to the experimental group will receive the same in-hospital care and physiotherapy prior to hospital discharge. They will also receive the same six-weekly, group-based 30-minute exercise session. The only difference between the two groups is the addition of a 30-minute group-based (up to 12 people) behaviour change approach intervention prior to the routine 30 minutes of exercise and three telephone-follow-up calls two, four and six weeks after the last group-based session (the PEP-TALK intervention).

The PEP-TALK intervention is theoretically-based within the cognitive-behavioural model of understanding. It uses evidence-based behaviour change techniques to target internal (cognitions and behaviours regarding physical activity) and external factors (social and environmental barriers). It specifically aims to target self-efficacy beliefs and fear-avoidant behaviours. A senior health psychologist (BF), physiotherapist/cognitive-behavioural therapist (ZH) and senior clinical academic physiotherapist (TS) developed an in-person, one-day training session. The training was delivered by BF and TS to all physiotherapists in the trial who were delegated to deliver the PEP-TALK intervention. The training included role-play, knowledge and understanding testing and supplementary materials to support on-going learning. All physiotherapists were invited to contact the training team (BF, ZH,TS) to clarify any training queries throughout the trial.

In the PEP-TALK sessions, through group discussion, participants and physiotherapists will be encouraged to develop a positive therapeutic alliance, where the physiotherapist will generate an environment of trust and belief around individual challenges participant's have, to support them to overcome these for sustained physical activity adoption. A treatment log will be completed by the physiotherapists to record the components of what is discussed across the group in each session.

For peer review only - http://bmjopen.bmj.com/site/about/guidelines.xhtml

The PEP-TALK intervention also includes a home-practice element. Participants will be supported with skills developed in the group, to work at home on challenges, barriers and facilitators to physical activity behaviour. The 'home-work' workbook aims to translate and develop the skills learnt within the group session into daily life activities. The workbook is designed to be used during the six sessions although participants are encouraged to keep this as a record and prompt for long-term behaviour change. The workbook will be referred to in the telephone follow-up calls. The workbook encourages reflective activities such as recording physical, emotional and cognitive barriers and facilitators to physical activity. It also offers problem-solving techniques such as "can you break down a large physical activity (e.g. 'getting the house all cleaned for the family coming over') into tasks which you can 'prioritise', 'plan', 'tolerance level' and 'evaluate.'" The workbook also includes other activities to encourage pacing and behaviour modification, goal-setting to the individual's health and social needs and techniques to challenge fear-avoidant behaviours. Education on exercise and the detrimental effects of physical inactivity will also be discussed. Participants will complete this with their home exercise plan which solely focuses on lower limb exercises rather than their behaviours and thoughts around physical activity.

The same trained physiotherapists who deliver the PEP-TALK intervention will, once the participant has completed their group sessions, deliver three, 20-minute telephone follow-up calls. These will occur two, four and six weeks after completing the group sessions. During these follow-up telephone calls, participant's goals will be reviewed, any barriers to the completion of these goals will be identified, and the physiotherapist will review any 'unhelpful' and 'helpful' thoughts or feelings towards physical activity which may have arisen since the last consultation. Each telephone call will close with the development of longer-term physical activity goal-setting and promotion of empowerment towards physical activity participation using the behavioural principles instilled during the group intervention. A log will be collected by the physiotherapist to record the components of what is discussed during the telephone calls.

Delivery

Both the PEP-TALK intervention and exercise groups will be delivered as 'rolling' programmes. This means that new participants can join the group as it runs rather than waiting for a new 'block' of sessions to start. This will allow greater flexibility in allocating participants to join group sessions, whilst also avoiding a delay between post-operative referral and starting the sessions. The flexible nature of the experimental intervention also makes this feasible, with no ordering of the content of intervention sessions which would preclude such a rolling programme.

Contamination

The physiotherapists who deliver the behaviour change sessions will be taught the skills required to deliver the experimental intervention. These physiotherapists will not be permitted to deliver the control exercise group intervention during the trial period (and vice versa). This approach mitigates the risk of contamination. Due to the interventions being delivered in an out-patient setting, there is a reduced risk of participants sharing their knowledge and experience of the interventions between the control and intervention groups, further minimising the risk of between-group contamination. Participants in the control group will not be permitted to join the experimental intervention group's exercise sessions (and vice versa). This will also reduce the risk of between-group contamination.

Co-Interventions

During the course of follow-up, participants may require further interventions as part of their recovery following surgery as per routine NHS practice. Further clinical interventions will be

For peer review only - http://bmjopen.bmj.com/site/about/guidelines.xhtml

permitted for trial participants without the participant having to withdraw. If a participant receives additional treatment to the trial intervention, the details of the treatment received and the reasons will be collected.

Quality Assessment

The trial will be monitored and audited in accordance with the current approved protocol, good clinical practice,[24] relevant regulations and standard operating procedures (SOPs).

All designated physiotherapists who deliver the usual care group exercises will be taught about the standardised control intervention procedures, i.e. clarification on use of exercise diaries and treatment logs which are additional to usual care.

Designated physiotherapists delivering the behaviour change intervention will attend a one-day, face-to-face course where they will be taught the intervention and processes involved by a member of the PEP-TALK team who developed the intervention (BF, ZH, TS). In addition, to assess the fidelity to the trial intervention, a health psychologist (BF) will undertake a debriefing telephone call with each physiotherapist after they have delivered their first group session. This will allow the trainer to re-enforce any learning required, address any uncertainties and to identify and correct any variation in the treatment protocol. Finally, each physiotherapist who delivers either the PEP-TALK or control group intervention will be monitored during a site visit at their 3rd or 4th intervention session. The PEP-TALK intervention physiotherapist will be monitored by a health psychologist (BF) or physiotherapist with expertise in health psychology (ZH). Control group interventions will be monitored by a practicing physiotherapist (TS). Sessions will be monitored against the protocol to determine whether there are issues around fidelity, contamination across groups or adherence/compliance of participants. Where further training is required, this will be instigated following these visits. Further monitoring visits will be co-ordinated as required.

Assessments

Baseline Assessment

Baseline data will be collected prior to randomisation during the pre-operative assessment appointment, once consent has been obtained. This will include: gender, age, measured height and weight, CCI, self-reported presence and location of multi-site joint pain, co-morbidities determined from the medical notes, AMTS, employment status and occupation (when appropriate). Participants will also complete measures on: physical activity (University of California Los Angeles (UCLA) Activity Scale;[25] physical function (Lower Extremity Functional Scale (LEFS);[26] disease-specific function (Oxford Hip Score [27] or Oxford Knee Score [28]); pain (numerical rating scale for pain); self-efficacy (Generalized Self-Efficacy Scale [29]); fear of movement or kinesiophobia (Tampa Scale for Kinesiophobia [30]); psychological distress (Hospital Anxiety and Depression Scale (HADS) [31]); HRQoL (EQ-5D-5L [32]) and the health utilisation questionnaire.

Follow-up data will be collected six- and 12-months post-randomisation. Questionnaires will be sent to participants by post from the trial office and returned using a pre-addressed, pre-paid envelope. If participants have not responded within 14 days of posting, the trial team will attempt to telephone the participant on up to two occasions to remind them to complete the questionnaires. If required, a second postage of the questionnaires will be provided if requested by the participant during these follow-up telephone calls. If participants wish not to complete the questionnaires, they will be

For peer review only - http://bmjopen.bmj.com/site/about/guidelines.xhtml

provided with the opportunity to complete the UCLA Activity Scale and EQ-5D-5L questionnaires over the telephone. If these methods fail, the participant would be categorised as a non-responder for that time-point only.

In an effort to find evidence-based techniques to improve retention and recruitment to RCTs, a study within a trial (SWAT) will be undertaken. This will examine whether there is a difference in questionnaire response rate by printing the UCLA Activity Scale on pink rather than white coloured paper at the six-month time-point. Results of this SWAT will be reported separately to the 'host' trial.

Outcome Measures

The data collection schedule is presented in **Table 2.**

Primary Outcome

- UCLA Activity Scale.[25] This is a reliable and valid self-reported tool to assess physical activity.[33,34] It assesses global activity levels with a grading system of one out of 10 points where one equates to "wholly inactive, dependent on others, and cannot leave residence" and 10 refers to "regularly participants in impact sports".[25]

Secondary Outcomes

- LEFS.[26] This is a valid and reliable measure of functional impairment in patients with lower extremity muscloskeletal condictions (Mehta et al, 2016).

- Oxford Hip Score (OHS) [27] or Oxford Knee Score (OKS).[28] Both measures have demonstrated good validity and reliability for people undergoing THR or TKR.[35]

- Numerical Rating Scale (NRS) for Pain. The NRS has been previously reported as reliable and valid to assess pain.[36]

- Generalized Self-Efficacy Scale.[29] This is a reliable and valid measure of self-efficacy.[37,38].

- The Tampa Scale for Kinesiophobia.[30] This is a valid and reliable measure of kinesiophobia.[39,40]

- HADS.[31] This has been shown as a reliable and valid measure of anxiety and depression.[41]

- Complications or adverse events (self-reported) which may include: wound or joint infection, joint dislocation, delayed hospital discharge (measured by length of stay), falls or musculoskeletal injuries and exacerbations of multi-site joint pain. Adverse events and serious adverse events will be reported as per the clinical trial unit's SOP.

- Health economic and health resource utilisation. We will collect HRQoL using the EQ-5D-5L.[33] Primary sources (i.e. participant log books) will be used to record the duration of the assessment visits, number and duration of scheduled group sessions, equipment,

For peer review only - http://bmjopen.bmj.com/site/about/guidelines.xhtml

consumables, educational material, behaviour change intervention training time; number and duration of scheduled telephone calls. Frequency of health care resource use will be collected through patient self-reported questionnaires at follow-ups. The categories of NHS resource use that will be collected include treatment costs, outpatient visits, any related-inpatient admissions, rehabilitation visits and any treatment for recurrences and adverse events. Non-medical costs such as caregiving and productivity loss for those in employment at each follow-up will be collected.

Data Analysis

Sample Size

Originally, 250 participants (125 per arm) are required to detect a standardised effect size of 0.4 with 80% power and 5% (two-sided) significance, and allowing for 20% loss to follow-up. These calculations are based on the primary outcome, UCLA Activity Scale, at 12 months, assuming a baseline standard deviation of 2.5 and a between-group difference of one.[33] The minimally clinically important difference (MCID) has been reported as a within person difference of 0.92 points.[33]

The sample size was increased to 260 to account for the amendment in randomisation proceeedure from 1:1 to 2:1 group allocation, as described earlier.

Statistical Analysis

A detailed statistical analysis plan (SAP) will be drafted early in the trial and will be finalised prior to any primary outcome analysis. This will be reviewed and will receive input from the Trial Steering Committee (TSC) and the DSMC.

All analyses will be undertaken on the intent-to-treat population, i.e. patients will be analysed as they were randomised regardless of the treatment received. Sensitivity analyses will be undertaken on the per-protocol population to assess a range of potential biases that could have resulted from loss-to-follow-up, protocol deviations and withdrawal (including mortality). Numerical and graphical summaries of all data will be presented including descriptions of missing data at each level.

Estimates of treatment effects will be reported with 95% confidence intervals. The primary outcome measure, UCLA Activity Scale at 12 months post-randomisation, will be analysed using a mixed-effects linear regression model adjusted for baseline activity, six month time point and the stratification factors. Centre will be included as a random-effect to take account of their potential heterogeneity and type of operation, comorbidity index, and other time points (baseline and six months) will be included as fixed-effects. Treatment by six month time point interaction will also be included in the model to allow time specific treatment effects to be calculated The adjusted difference in the means with corresponding 95% confidence interval will be reported together with the mean and 95% confidence intervals for each treatment group. If not normally distributed, then transformation to normality will be considered. If this is not possible, non-parametric techniques (for example Mann-Whitney or Kruskal-Wallis Test) will be used with no adjustment. Consistency of any observed intervention effect will be explored using forest plots for various subgroups including site, type of operation (TKR and THR) and comorbidity index using interactions. No formal testing of interaction effects will be undertaken as the trial is not powered to detect these and the effect of

For peer review only - http://bmjopen.bmj.com/site/about/guidelines.xhtml

For peer review only

the intervention is expected to be similar in the THR and TKR subgroups, although their baseline physical activity may be different, which will be taken into account in the analysis.

Similar methods will be used to analyse continuous secondary outcomes. Binary and categorical secondary outcomes, such as complications, will be tabulated to show frequencies and percentages in each arm. Chi-squared tests or logistic regression will be used to assess statistical significance.

Missing data will be minimised by careful data management. Missing data will be described with reasons given where available. The number and percentage of individuals in the missing category will be presented by treatment arm.

The nature and mechanism for missing variables and outcomes will be investigated, and if appropriate, multiple imputation will be used. Sensitivity analyses will be undertaken assessing the underlying missing data assumptions. Any imputation techniques will be fully described in the SAP. Subgroup analyses, complier average causal effect (CACE) analysis and mediation analyses will be undertaken after being fully specified in the SAP. Compliance with the intervention is defined as attending at least four of six group sessions with a minimum of three participants in the group for these sessions and receiving at least two of the three follow-up phone calls. A priori mediation analysis moderators will include self-efficacy, fear avoidance, psychological distress to compare the mediation pathways presented in the BeST intervention [42] to the PEP-TALK intervention.

All data collected on data collection forms will be used, since only essential data items will be collected. No data will be considered spurious in the analysis since all data will be checked and cleaned before analysis.

<u>Health Economics</u>

Respecting an *efficient trial design*, only if the data indicates clinical effectiveness of the experimental intervention (for the UCLA Activity Scale and LEFS), will additional funding be sought to analyse the health economic and utilisation data, to determine the cost-effectiveness of the intervention.

<u>Data Management</u>

All data will be processed according to the Data Protection Act 2018.[43] All documents will be stored safely in confidential conditions. Trial-specific documents, except for the signed consent form and follow-up contact details, will refer to the participant with a unique study participant number, not by name. Participant identifiable data will be stored separately from trial data. All trial data will be stored securely in offices or online in secure trial databases, only accessible by the central trial team in Oxford and authorised personnel.

Trial Status

The trial is funded for 36 months and commenced in August 2018. Recruitment is expected to be complete by April 2020 with the final follow-up visit for the final participant completed by April 2021. The trial will be completed by August 2021.

For peer review only - http://bmjopen.bmj.com/site/about/guidelines.xhtml

Site Locations

Orthopaedic services providing THR and TKRs in nine hospital trusts (eight recruiting sites) and health providers: (Norfolk and Norwich University Hospitals NHS Foundation Trust, Lewisham and Greenwich University Hospitals NHS Foundation Trust, Oxford University Hospital NHS Foundation Trust, City Hospitals Sunderland NHS Foundation Trust, Norwich Spire, Barts Health NHS Trust, North Middlesex University Hospital NHS Trust, St George's University Hospital NHS Foundation Trust (treatment site only), Epsom and St Helier University Hospitals NHS Foundation Trust (recruitment site only)).

Patient and Public Involvement

Patient involvement began during protocol development and continues throughout the trial. A patient-member will attend all TSC meetings. The same patient-member is a co-investigator, providing insights into the trial conduct, particularly on data collection processes, and will help interpret the findings to inform on the implications of the research during the trial's dissemination phase.

ETHICS AND DISSEMINATION

Ethical approval was gained from the South Central (Oxford B) Research Ethics Committee (Approval Date: 23Oct2018; Reference Number: 18/SC/0423). The trial was prospectively registered (ISRCTN29770908). A DSMC and TSC was appointed to independently review the data on safety, protocol adherence and recruitment to the trial. Direct access will be granted to authorised representatives from the sponsor and host institution for monitoring and/or audit of the trial to ensure compliance with regulations. Annoymised data will be shared outside the research team when required. Researchers outside the trial team may formally request for a specific data set using a data request form, which will be part of the Data Management Plan (DMP). All such requests will need to be approved by the TMG.

Reporting of the trial will be consistent with the CONSORT 2010 Statement (patient reported outcomes and non-pharmcological interventions)[44] and Template for Intervention Description and Replication (TIDieR)[45] guidelines. A summary of the results and trial materials will be made available via the trial website on completion of the trial. We will submit the final report to a peer-reviewed academic journal.

DISCUSSION

This paper presents the research protocol for the PEP-TALK trial. Following a THR and TKR, only 50% of people reach World Health Organization recommended levels of physical activity.[15,46,47] Those who are least likely to meet these levels are people with a higher body mass index and with comorbidities.[48] These patients have the most to gain from being more physically active. If this trial's experimental intervention is shown to be effective, this intervention could have a significant and sustained impact on improving the management of comorbidities such as diabetes, cardiovascular diseases, depression and hypertension for over 103,000 people annually in England

For peer review only - http://bmjopen.bmj.com/site/about/guidelines.xhtml

and Wales.[1] It is proposed that, in such an instance, this should be considered for implementation in healthcare services. This could help address the global challenge which multi-morbidities and an ageing population are expected to have on health and social care services.

DECLARATIONS AND ACKNOWLEDGEMENTS

Authors' Contributors: TS, BF, SD, SL, AO, CH, VB, SP researched the topic and devised the study. TS, SP, AO, BF, SD, CH, VB, MEP, SL provided the first draft of the manuscript. AO, SD provided statistical oversight. TS, SP, BF, AO, SD, CH, VB, MEP, SL contributed equally to manuscript preparation. TS acts a guarantor.

Funding Statement: The research is supported by the National Institute for Health Research (NIHR) Research for Patient Benefit grant (PB-PG-1216-20008). The views expressed are those of the author(s) and not necessarily those of the NHS, the NIHR or the Department of Health and Social Care.

Competing Interests Statement: None declared.

Disclaimer: TS is supported by the National Institute for Health Research (NIHR) Oxford Biomedical Research Centre (BRC). The views expressed are those of the author(s) and not necessarily those of the NHS, the NIHR or the Department of Health and Social Care.

Ethics Approval: Ethical approval was gained from the South Central (Oxford B) Research Ethics Committee (Approval Date: 23Oct2018; Reference Number: 18/SC/0423).

Trial Sponsor: University of Oxford (Clinical Trials and Research Governance Team. Joint Research Office, 1st floor, Boundary Brook House, Churchill Drive, Headington, Oxford OX3 7GB. Email: ctrg@admin.ox.ac.uk. The views expressed are those of the author(s) and not necessarily those of the sponsor.

Provenance and Peer Review: Not commissioned; externally peer reviewed.

PEP-TALK Collaborators: Mr Steve Algar (Banbury, PPI Representative), Dr Zara Hansen (ZH) (University of Oxford), Professor Karen Barker (Principal Investigator - Oxford University Hospital NHS Foundation Trust (OUH)**,** Mr Ian Smith (Principal Investigator **-** Lewisham and Greenwich University Hospitals NHS Foundation Trust), Professor Iain McNamara (Principal Investigator (Norfolk and Norwich University Hospitals NHS Foundation Trust (NNUH) & Spire Norwich), Mr Michael Dunn (Principal Investigator - St George's University Hospital NHS Foundation Trust), Mrs Dawn Lockey (Principal Investigator - City Hospitals Sunderland NHS Foundation Trust), Mr Sonny Driver (Principal Investigator – North Middlesex University Hospital NHS Trust), Mrs Jamila Kassam (Barts Health NHS Trust), Mr Peter Penny (Norwich Spire, Norwich), Mrs Celia Woodhouse, Mrs Tracey Potter and Mrs Helena Daniell (NNUH), Mr Alex Herring and Mrs Yan Cunningham (City Hospitals Sunderland NHS Foundation Trust), Irrum Afzal (South West London Elective Orthopaedic Centre), Mr Maninderpal Matharu (Barts Health NHS Trust) and Mrs Tamsin Hughes, Ms Erin Hannink and Mrs Michelle Moynihan (OUH).

Oversight Committee Membership: TSC Members: Professor David Deehan (Newcastle University), Dr Emma Godfrey (Kings College London), Dr Neil Artz (University of the West of England), Mr Steve Algar (PPI representative). **DSMC Members:** Dr Lindsey Smith (University of the West of England &

For peer review only - http://bmjopen.bmj.com/site/about/guidelines.xhtml

Weston Area Health NHS Trust), Dr Dipesh Mistry (University of Warwick), Mr Paul Baker (South Tees Hospital NHS Foundation Trust)

Provenance and peer review: Not commissioned; externally peer reviewed.

Patient consent for publication: Not required.

Word count: 4678

For peer review only

For peer review only - http://bmjopen.bmj.com/site/about/guidelines.xhtml

FIGURE AND TABLE LEGENDS

Figure 1: Study flow chart

Table 1: PEP-TALK trial objectives, outcome measures and measurement time-points

Table 2: Data collection schedule

Supplementary File 1: Consent form

REFERENCES

1. National Joint Registry. 12th Annual Report. Accessed 15.09.2016. Available at: http://www.njrcentre.org.uk/njrcentre/Portals/0/Documents/England/Reports/12th%20annual%20eport/NJR%20Online%20Annual%20Report%202015.pdf

2. Dailiana ZH, Papakostidou I, Varitimidis S, Liaropoulos L, Zintzaras E, Karachalios T, Michelinakis E, Malizos KN. Patient-reported quality of life after primary major joint arthroplasty: a prospective comparison of hip and knee arthroplasty. BMC Musculoskelet Disord. 2015 Nov 26;16:366.

3. Papakostidou I, Dailiana ZH, Papapolychroniou T, Liaropoulos L, Zintzaras E, Karachalios TS, Malizos KN. Factors affecting the quality of life after total knee arthroplasties: a prospective study. BMC Musculoskelet Disord. 2012 Jun 29;13:116.

4. Withers TM, Lister S, Sackley C, Clark A, Smith TO. Is there a difference in physical activity levels in patients before and up to one year after unilateral total hip replacement? A systematic review and meta-analysis. Clin Rehabil. 2016: In Press.

5. Smith TO, Hilton G, Mansfield M, Mann CJV, Sackley CM. Does physical activity change following hip and knee arthroplasty? An analysis of data from the Osteoarthritis Initiative. Physiotherapy. 2018; In Press.

6. Smith TO, Latham S, Maskrey V, Blyth A. Patients' perceptions of physical activity before and after joint replacement: a systematic review with meta-ethnographic analysis. Postgrad Med J. 2015 Sep;91(1079):483-91.

7. Inacio MC, Pratt NL, Roughead EE, Graves SE. Comparing co-morbidities in total joint arthroplasty patients using the RxRisk-V, Elixhauser, and Charlson Measures: a cross-sectional evaluation. BMC Musculoskelet Disord. 2015 Dec 10;16:385.

8. Peter WF, Dekker J, Tilbury C, Tordoir RL, Verdegaal SH, Onstenk R, Bénard MR, Vehmeijer SB, Fiocco M, Vermeulen HM, van der Linden-van der Zwaag HM, Nelissen RG, Vliet Vlieland TP. The association between comorbidities and pain, physical function and quality of life following hip and knee arthroplasty. Rheumatol Int. 2015 Jul;35(7):1233-41.

For peer review only

For peer review only - http://bmjopen.bmj.com/site/about/guidelines.xhtml

9. Smith E, Hoy DG, Cross M, Vos T, Naghavi M, Buchbinder R, Woolf AD, March L. PICO. The global burden of other musculoskeletal disorders: estimates from the Global Burden of Disease 2010 study. Ann Rheum Dis. 2014 Aug;73(8):1462-9.

10. Sampson UK, Fowkes FG, McDermott MM, Criqui MH, Aboyans V, Norman PE, Forouzanfar MH, Naghavi M, Song Y, Harrell FE Jr, Denenberg JO, Mensah GA, Ezzati M, Murray C. Global and regional burden of death and disability from peripheral artery disease: 21 world regions, 1990 to 2010. Glob Heart. 2014 Mar;9(1):145-158.e21.

11. Lee CD, Folsom AR, Blair SN. Physical activity and stroke risk: a meta-analysis. Stroke. 2003 Oct;34(10):2475-81.

12. Laaksonen DE, Lindström J, Lakka TA, Eriksson JG, Niskanen L, Wikström K, Aunola S, Keinänen-Kiukaanniemi S, Laakso M, Valle TT, Ilanne-Parikka P, Louheranta A, Hämäläinen H, Rastas M, Salminen V, Cepaitis Z, Hakumäki M, Kaikkonen H, Härkönen P, Sundvall J, Tuomilehto J, Uusitupa M; Finnish diabetes prevention study. Physical activity in the prevention of type 2 diabetes: the Finnish diabetes prevention study. Diabetes. 2005 Jan;54(1):158-65.

13. Lee IM. Physical activity and cancer prevention--data from epidemiologic studies. Med Sci Sports Exerc. 2003 Nov;35(11):1823-7.

14. Nocon M, Hiemann T, Müller-Riemenschneider F, Thalau F, Roll S, Willich SN. Association of physical activity with all-cause and cardiovascular mortality: a systematic review and meta-analysis. Eur J Cardiovasc Prev Rehabil. 2008 Jun;15(3):239-46.

15. Kersten RF, Stevens M, van Raay JJ, Bulstra SK, van den Akker-Scheek I. Habitual physical activity after total knee replacement. Phys Ther. 2012 Sep;92(9):1109-16.

16. French DP, Olander EK, Chisholm A, Mc Sharry J. Which behaviour change techniques are most effective at increasing older adults' self-efficacy and physical activity behaviour? A systematic review. Ann Behav Med. 2014 Oct;48(2):225-34.

17. Devereux-Fitzgerald A, Powell R, Dewhurst A, French DP. The acceptability of physical activity interventions to older adults: A systematic review and meta-synthesis. Soc Sci Med. 2016 Jun;158:14-23.

18. Friederichs SA, Oenema A, Bolman C, Lechner L. Long term effects of self-determination theory and motivational interviewing in a web-based physical activity intervention: randomized controlled trial. Int J Behav Nutr Phys Act. 2015 Aug 18;12:101.

19. Department of Health. 2013. General Practice Physical Activity Questionnaire. Accessed on 21.02.2017. Available at: https://www.gov.uk/government/publications/general-practice-physical-activity-questionnaire-gppaq

20. Charlson ME, Pompei P, Ales KL, MacKenzie CR. A new method of classifying prognostic comorbidity in longitudinal studies: development and validation. J Chronic Dis. 1987;40(5):373-83.

21. Charlson ME, Charlson RE, Briggs W, Hollenberg J. Can disease management target patients most likely to generate high costs? The impact of comorbidity. J Gen Intern Med. 2007; 22: 464-469.

For peer review only - http://bmjopen.bmj.com/site/about/guidelines.xhtml

22. Hodkinson HM. Evaluation of a mental test score for assessment of mental impairment in the elderly. Age Ageing 1972;1: 233–8.

23. Briggs TWR. 2012. Getting it right first time. Improving the quality of orthopaedic care within the National Health Service in England. Accessed 06.10.2016. Available at: http://www.gettingitrightfirsttime.com/downloads/briggsreporta4_fin.pdf

24. International Conference on Harmonisation Good Clinical Practice (ICH GCP). International Conference on Harmonisation Good Clinical Practice Recommendations. Accessed: 08 March 2018. Available at: https://www.ich.org/fileadmin/Public_Web_Site/ICH_Products/Guidelines/Efficacy/E6/E6_R1_Guideline.pdf

25. Zahiri CA, Schmalzried TP, Szuszczewicz ES, Amstutz HC. Assessing activity in joint replacement patients. J Arthroplasty. 1998;13:890–895

26. Binkley JM, Stratford OW, Lott SA, Riddle DL. The Lower Extremity Functional Scale (LEFS): Scale development, measurement properties, and clinical application. Physical Therapy 1999;79:371-383

27. Dawson J, Fitzpatrick R, Carr A, Murray D. Questionnaire on the perceptions of patients about total hip replacement. J Bone Joint Surg Br. 1996 Mar;78(2):185-90.

28. Dawson J, Fitzpatrick R, Murray D, Carr A. Questionnaire on the perceptions of patients about total knee replacement. J Bone Joint Surg Br. 1998 Jan;80(1):63-9.

29. Schwarzer R, Jerusalem M. Generalized Self-Efficacy scale. In J. Weinman, S. Wright, & M. Johnston, Measures in health psychology: A user's portfolio. Causal and control beliefs 1995, pp. 35-37.

30. Miller RP, Kori S, Todd D. The Tampa Scale: a measure of kinesiophobia. Clin J Pain 1991;7(1):51–52.

31. Zigmond AS, Snaith RP. The hospital anxiety and depression scale. Acta Psychiatrica Scandinavica. 1983; 67: 361–370.

32. EuroQol Group E (1990) EuroQol—a new facility for the measurement of health-related quality of life. Health Policy 16: 199–208.

33. SooHoo NF, Li Z, Chenok KE, Bozic KJ. Responsiveness of patient reported outcome measures in total joint arthroplasty patients. J Arthroplasty. 2015 Feb;30(2):176-91.

34. Naal FD, Impellizzeri FM, Leunig M. Which is the best activity rating scale for patients undergoing total joint arthroplasty? Clin Orthop Relat Res. 2009 Apr;467(4):958-65.

35. Harris K, Dawson J, Gibbons E, Lim CR, Beard DJ, Fitzpatrick R, Price AJ. Systematic review of measurement properties of patient-reported outcome measures used in patients undergoing hip and knee arthroplasty. Patient Relat Outcome Meas. 2016 Jul 25;7:101-8. doi: 10.2147/PROM.S97774.

For peer review only - http://bmjopen.bmj.com/site/about/guidelines.xhtml

36. Ferreira-Valente MA, Pais-Ribeiro JL, Jensen MP. Validity of four pain intensity rating scales. Pain. 2011 Oct;152(10):2399-404.

37. Bonsaksen T, Kottorp A, Gay C, Fagermoen MS, Lerdal A. Rasch analysis of the General Self-Efficacy Scale in a sample of persons with morbid obesity. Health Qual Life Outcomes. 2013 Nov 25;11:202. doi: 10.1186/1477-7525-11-202.

38. Barlow JH, Williams B, Wright C. The Generalized Self-Efficacy Scale in people with arthritis. Arthritis Care Res. 1996 Jun;9(3):189-96.

39. Lundberg MKE, Styf J, Carlsson SG. A psychometric evaluation of the Tampa Scale for Kinesiophobia – from a physiotherapeutic perspective. Physiotherapy Theory and Practice. 2004;20(2):121–133.

40. Bunketorp L, Carlsson J, Kowalski J, Stener-Victorin E. Evaluating the reliability of multi-item scales: a non-parametric approach to the ordered categorical structure of data collected with the Swedish version of the Tampa Scale for Kinesiophobia and the Self-Efficacy Scale. J Rehabil Med. 2005 Sep;37(5):330-4

41. Bjelland I, Dahl AA, Haug TT, Neckelmann D. The validity of the Hospital Anxiety and Depression Scale: an updated literature review. J Psychosom Res. 2002;52(2):69–77

42. Lamb SE, Lall R, Hansen Z, Castelnuovo E, Withers EJ, Nichols V, Griffiths F, Potter R, Szczepura A, Underwood M; BeST trial group. A multicentred randomised controlled trial of a primary care-based cognitive behavioural programme for low back pain. The Back Skills Training (BeST) trial. Health Technol Assess. 2010 Aug;14(41):1-253, iii-iv.

43. Data Protection Act. 2018. Accessed: 30 September 2019. Available at: http://www.legislation.gov.uk/ukpga/2018/12/contents/enacted

44. Schulz KF , Altman DG , Moher D , et al. CONSORT 2010 statement: updated guidelines for reporting parallel group randomised trials. BMC Med 2010;8:18

45. Hoffmann TC , Glasziou PP, Boutron I, et al. Better reporting of interventions: template for intervention description and replication (TIDieR) checklist and guide. BMJ 2014;348:g1687

46. Groen JW, Stevens M, Kersten RF, Reininga IH, van den Akker-Scheek I. After total knee arthroplasty, many people are not active enough to maintain their health and fitness: an observational study. J Physiother. 2012;58(2):113-6.

47. Wagenmakers R, Stevens M, Zijlstra W, Jacobs ML, van den Akker-Scheek I, Groothoff JW, Bulstra SK. Habitual physical activity behavior of patients after primary total hip arthroplasty. Phys Ther. 2008 Sep;88(9):1039-48.

48. Paxton EW, Torres A, Love RM, Barber TC, Sheth DS, Inacio MC. Total joint replacement: A multiple risk factor analysis of physical activity level 1-2 years postoperatively. Acta Orthop. 2016 Jul;87 Suppl 1:44-9.

For peer review only

1 **Table 1:** PEP-TALK trial objectives, outcome measures and measurement time-points

Objectives	Outcome Measures	Time-points
**Primary Objective**		
To compare physical activity participation at 12 months post randomisation between people who receive a group exercise and behaviour change intervention versus group exercise alone following THR or TKR.	University of California, Los Angeles (UCLA) activity scale	Baseline; 6 months; 12 months (primary time-point)
**Secondary Objectives**		
To compare the functional outcome between people who receive a group exercise and behaviour-change intervention versus group exercise alone following THR or TKR.	Lower Extremity Functional Scale (LEFS)	Baseline; 6 months; 12 months
To compare the disease-specific pain and function between people who receive a group exercise and behaviour-change intervention versus group exercise alone following THR or TKR.	Oxford Hip Score (OHS) [35] or Oxford Knee Score (OKS)	Baseline; 6 months; 12 months
To compare the perceived level of pain between people who receive a group exercise and behaviour-change intervention versus group exercise alone following THR or TKR.	Numerical Rating Scale (NRS) for Pain	Baseline; 6 months; 12 months
To compare participant's self-efficacy between those who receive a group exercise and behaviour-change intervention versus group exercise alone following THR or TKR.	Generalized Self-Efficacy Scale (GSES)	Baseline; 6 months; 12 months
To compare participant's fear avoidance to movement between those who receive a group exercise and behaviour-change intervention versus group exercise alone following THR or TKR.	The Tampa Scale for Kinesiophobia	Baseline; 6 months; 12 months
To compare participant's psychological distress (anxiety and depression) between those who receive a group exercise and behaviour-change intervention versus group exercise alone following THR or TKR.	Hospital Anxiety and Depression Scale (HADS)	Baseline; 6 months; 12 months
To compare participant's complications and adverse events between those who receive a group exercise and behaviour-change intervention versus group exercise alone following THR or TKR.	Complications or adverse events recorded in the CRFs	6 months; 12 months
To collect cost-effectiveness data (health resource utilisation; direct and indirect costs) of a group exercise and a behaviour-change intervention versus group exercise alone following THR or TKR.	EQ-5D-5L; Bespoke health care resource self-reported questionnaire	6 months; 12 months

2

For peer review only - http://bmjopen.bmj.com/site/about/guidelines.xhtml

Table 2: Data collection schedule

<table>
<tr><th rowspan="3">Data</th><th colspan="4">Time-points and Mode of Data Collection</th></tr>
<tr><th>Baseline</th><th>Intervention Period</th><th>Month 6 Post-Randomisation</th><th>Month 12 Post-Randomisation</th></tr>
<tr><th>Face-to-Face</th><th>Face-to-Face</th><th>Postal</th><th>Postal</th></tr>
<tr><td>Age (years)</td><td style="background:#d9d9d9"></td><td></td><td></td><td></td></tr>
<tr><td>Gender</td><td style="background:#d9d9d9"></td><td></td><td></td><td></td></tr>
<tr><td>Weight (kg)</td><td style="background:#d9d9d9"></td><td></td><td></td><td></td></tr>
<tr><td>Height (cm)</td><td style="background:#d9d9d9"></td><td></td><td></td><td></td></tr>
<tr><td>Admission Date</td><td style="background:#d9d9d9"></td><td></td><td></td><td></td></tr>
<tr><td>Operative procedure (THR or TKR)</td><td style="background:#d9d9d9"></td><td></td><td></td><td></td></tr>
<tr><td>Side of Joint Replacement</td><td style="background:#d9d9d9"></td><td></td><td></td><td></td></tr>
<tr><td>Duration of hip or knee symptoms</td><td style="background:#d9d9d9"></td><td></td><td></td><td></td></tr>
<tr><td>Presence and location of multi-joint pain</td><td style="background:#d9d9d9"></td><td></td><td style="background:#d9d9d9"></td><td style="background:#d9d9d9"></td></tr>
<tr><td>ASA grade</td><td style="background:#d9d9d9"></td><td></td><td></td><td></td></tr>
<tr><td>AMTS</td><td style="background:#d9d9d9"></td><td></td><td></td><td></td></tr>
<tr><td>List of medical co-morbidities</td><td style="background:#d9d9d9"></td><td></td><td></td><td></td></tr>
<tr><td>Physiotherapist exercise class log</td><td></td><td style="background:#d9d9d9"></td><td></td><td></td></tr>
<tr><td>Patient in-session and home exercise diary</td><td></td><td style="background:#d9d9d9"></td><td></td><td></td></tr>
<tr><td>Behaviour change intervention log (group and telephone) (physiotherapist completed)</td><td></td><td style="background:#d9d9d9"></td><td></td><td></td></tr>
<tr><td>Charlson Comorbidity Score</td><td style="background:#d9d9d9"></td><td></td><td></td><td></td></tr>
<tr><td>Employment status and current occupation (when appropriate)</td><td style="background:#d9d9d9"></td><td></td><td style="background:#d9d9d9"></td><td style="background:#d9d9d9"></td></tr>
<tr><td>UCLA Activity Score</td><td style="background:#d9d9d9"></td><td></td><td style="background:#d9d9d9"></td><td style="background:#d9d9d9"></td></tr>
<tr><td>Lower Extremity Functional Scale</td><td style="background:#d9d9d9"></td><td></td><td style="background:#d9d9d9"></td><td style="background:#d9d9d9"></td></tr>
<tr><td>Oxford Hip or Knee Score</td><td style="background:#d9d9d9"></td><td></td><td style="background:#d9d9d9"></td><td style="background:#d9d9d9"></td></tr>
<tr><td>Numerical Rating Scale – Pain</td><td style="background:#d9d9d9"></td><td></td><td style="background:#d9d9d9"></td><td style="background:#d9d9d9"></td></tr>
<tr><td>Generalized Self-Efficacy Scale</td><td style="background:#d9d9d9"></td><td></td><td style="background:#d9d9d9"></td><td style="background:#d9d9d9"></td></tr>
<tr><td>Tampa Scale for Kinesiophobia</td><td style="background:#d9d9d9"></td><td></td><td style="background:#d9d9d9"></td><td style="background:#d9d9d9"></td></tr>
<tr><td>Hospital Anxiety and Depression Scale</td><td style="background:#d9d9d9"></td><td></td><td style="background:#d9d9d9"></td><td style="background:#d9d9d9"></td></tr>
<tr><td>EQ-5D-5L</td><td style="background:#d9d9d9"></td><td></td><td style="background:#d9d9d9"></td><td style="background:#d9d9d9"></td></tr>
<tr><td>Health economic/Health utilisation questionnaire</td><td></td><td></td><td style="background:#d9d9d9"></td><td style="background:#d9d9d9"></td></tr>
<tr><td>Complications and adverse events</td><td></td><td></td><td style="background:#d9d9d9"></td><td style="background:#d9d9d9"></td></tr>
</table>

Shaded areas represent where these data are being collected.

Assessment intervals following randomisation; each follow-up interval +/- 1 month

AMTS – Abbreviated Mental Test Score; ASA – American Society of Anaesthesiologists classification; cm – centimetres; kg – kilograms; THR – total hip replacement; TKR – total knee replacement; UCLA – University College Los Angeles.

For peer review only

For peer review only - http://bmjopen.bmj.com/site/about/guidelines.xhtml

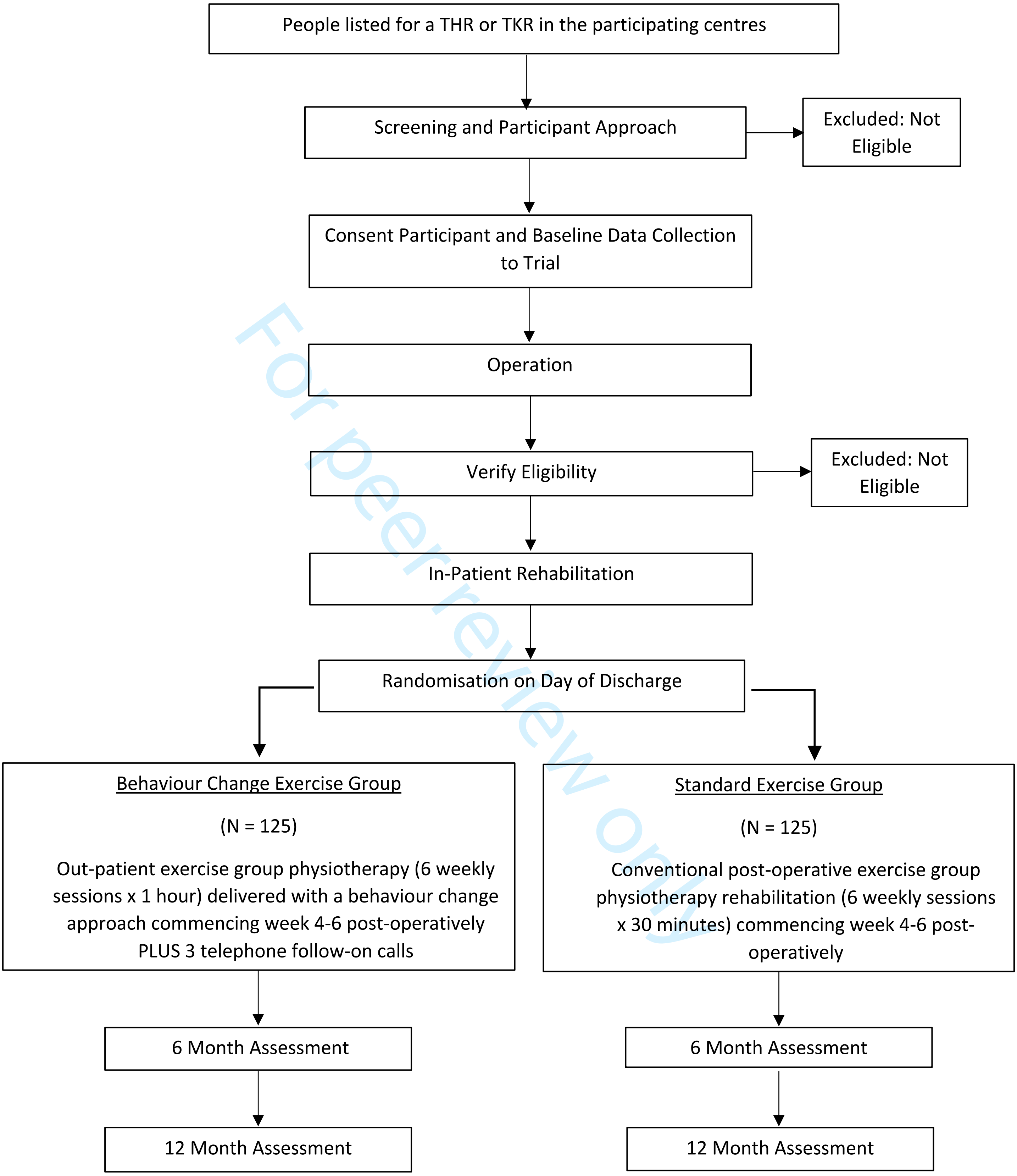

For peer review only - http://bmjopen.bmj.com/site/about/guidelines.xhtml

CONSENT FORM (PEP-TALK STUDY)

Name of Local Principal Investigator:___________________________________

LOCAL TRUST LOGO

Screening Number: ☐☐ - ☐☐☐☐

If you agree, please initial

1. I confirm that I have read and understood the Information Leaflet dated 10 October 2019 version 4.0. I have had the opportunity to consider the information, ask questions and have had these answered satisfactorily.	
2. I understand that my participation is voluntary and that I am free to withdraw at any time without giving any reason, and without my medical care or legal rights being affected.	
3. I understand that relevant sections of my medical notes and data collected during the study may be looked at by individuals from the University of Oxford, from regulatory authorities [and from the NHS Trust(s)], where it is relevant to me taking part in this research. I give permission for these individuals to have access to my records.	
4. I consent to the research team holding my contact details so that they can contact me about the study. I understand these details will be held securely and destroyed at the end of the study.	
5. I am aware that treatment sessions may be observed for quality assurance purposes.	
6. I agree to my General Practitioner (GP) being informed of my participation in the study and questionnaire results.	
7. I agree to be contacted for the purposes of follow up by the central PEP-TALK team who are based in Oxford.	
8. I agree to take part in the PEP-TALK study.	

**Name of Participant**	**Date**	**Signature**

**Name of Person Taking Consent**	**Date**	**Signature**

STUDY RESULTS – THIS SECTION IS OPTIONAL TO COMPLETE

I agree to be contacted about ethically approved research studies for which I may be suitable. I understand that agreeing to be contacted does not oblige me to participate in any further studies.

YES ☐ NO ☐

For peer review only

STANDARD PROTOCOL ITEMS: RECOMMENDATIONS FOR INTERVENTIONAL TRIALS

SPIRIT 2013 Checklist: Recommended items to address in a clinical trial protocol and related documents*

Section/item	Item No	Description	Location in Text
**Administrative information**			
Title	1	Descriptive title identifying the study design, population, interventions, and, if applicable, trial acronym	Title
Trial registration	2a	Trial identifier and registry name. If not yet registered, name of intended registry	Abstract
	2b	All items from the World Health Organization Trial Registration Data Set	Abstract to ID
Protocol version	3	Date and version identifier	Abstract page
Funding	4	Sources and types of financial, material, and other support	Abstract page & Declarations
Roles and responsibilities	5a	Names, affiliations, and roles of protocol contributors	Title Page & Declarations
	5b	Name and contact information for the trial sponsor	Declarations
	5c	Role of study sponsor and funders, if any, in study design; collection, management, analysis, and interpretation of data; writing of the report; and the decision to submit the report for publication, including whether they will have ultimate authority over any of these activities	Declarations

For peer review only - http://bmjopen.bmj.com/site/about/guidelines.xhtml

	5d	Composition, roles, and responsibilities of the coordinating centre, steering committee, endpoint adjudication committee, data management team, and other individuals or groups overseeing the trial, if applicable (see Item 21a for data monitoring committee)	Data Management and Ethics and Dissemination
**Introduction**			
Background and rationale	6a	Description of research question and justification for undertaking the trial, including summary of relevant studies (published and unpublished) examining benefits and harms for each intervention	Background, Para 5
	6b	Explanation for choice of comparators	BACkground Para 3-4; Methods, Interventions
Objectives	7	Specific objectives or hypotheses	Background, Para 4
Trial design	8	Description of trial design including type of trial (eg, parallel group, crossover, factorial, single group), allocation ratio, and framework (eg, superiority, equivalence, noninferiority, exploratory)	Methods, Trial Design
**Methods: Participants, interventions, and outcomes**			
Study setting	9	Description of study settings (eg, community clinic, academic hospital) and list of countries where data will be collected. Reference to where list of study sites can be obtained	Methods, Site Locations & Declarations
Eligibility criteria	10	Inclusion and exclusion criteria for participants. If applicable, eligibility criteria for study centres and individuals who will perform the interventions (eg, surgeons, psychotherapists)	Methods, Participants

For peer review only - http://bmjopen.bmj.com/site/about/guidelines.xhtml

Interventions	11a	Interventions for each group with sufficient detail to allow replication, including how and when they will be administered	Methods, Interventions
	11b	Criteria for discontinuing or modifying allocated interventions for a given trial participant (eg, drug dose change in response to harms, participant request, or improving/worsening disease)	Methods, Contamination
	11c	Strategies to improve adherence to intervention protocols, and any procedures for monitoring adherence (eg, drug tablet return, laboratory tests)	Methods, Quality Assessment
	11d	Relevant concomitant care and interventions that are permitted or prohibited during the trial	Methods, Cointerventions
Outcomes	12	Primary, secondary, and other outcomes, including the specific measurement variable (eg, systolic blood pressure), analysis metric (eg, change from baseline, final value, time to event), method of aggregation (eg, median, proportion), and time point for each outcome. Explanation of the clinical relevance of chosen efficacy and harm outcomes is strongly recommended	Methods, Outcome Measures & Table 2
Participant timeline	13	Time schedule of enrolment, interventions (including any run-ins and washouts), assessments, and visits for participants. A schematic diagram is highly recommended (see Figure)	Figure 1
Sample size	14	Estimated number of participants needed to achieve study objectives and how it was determined, including clinical and statistical assumptions supporting any sample size calculations	Data Analysis, Sample Size
Recruitment	15	Strategies for achieving adequate participant enrolment to reach target sample size	Methods, Trial Status, Site Locations
**Methods: Assignment of interventions (for controlled trials)**			
Allocation:			

Sequence generation	16a	Method of generating the allocation sequence (eg, computer-generated random numbers), and list of any factors for stratification. To reduce predictability of a random sequence, details of any planned restriction (eg, blocking) should be provided in a separate document that is unavailable to those who enrol participants or assign interventions	Methods, Randomisation, Blinding and Allocation
Allocation concealment mechanism	16b	Mechanism of implementing the allocation sequence (eg, central telephone; sequentially numbered, opaque, sealed envelopes), describing any steps to conceal the sequence until interventions are assigned	Methods, Randomisation, Blinding and Allocation
Implementation	16c	Who will generate the allocation sequence, who will enrol participants, and who will assign participants to interventions	Methods, Randomisation, Blinding and Allocation
Blinding (masking)	17a	Who will be blinded after assignment to interventions (eg, trial participants, care providers, outcome assessors, data analysts), and how	Methods, Randomisation, Blinding and Allocation
	17b	If blinded, circumstances under which unblinding is permissible, and procedure for revealing a participant's allocated intervention during the trial	Methods, Randomisation, Blinding and Allocation
**Methods: Data collection, management, and analysis**			
Data collection methods	18a	Plans for assessment and collection of outcome, baseline, and other trial data, including any related processes to promote data quality (eg, duplicate measurements, training of assessors) and a description of study instruments (eg, questionnaires, laboratory tests) along with their reliability and validity, if known. Reference to where data collection forms can be found, if not in the protocol	Methods, Outcome Measures & Table 2

For peer review only - http://bmjopen.bmj.com/site/about/guidelines.xhtml

	18b	Plans to promote participant retention and complete follow-up, including list of any outcome data to be collected for participants who discontinue or deviate from intervention protocols	Methods, Assessments
Data management	19	Plans for data entry, coding, security, and storage, including any related processes to promote data quality (eg, double data entry; range checks for data values). Reference to where details of data management procedures can be found, if not in the protocol	Methods, Assessment & Data Management
Statistical methods	20a	Statistical methods for analysing primary and secondary outcomes. Reference to where other details of the statistical analysis plan can be found, if not in the protocol	Data Analysis
	20b	Methods for any additional analyses (eg, subgroup and adjusted analyses)	Data Analysis
	20c	Definition of analysis population relating to protocol non-adherence (eg, as randomised analysis), and any statistical methods to handle missing data (eg, multiple imputation)	Data Analysis
**Methods: Monitoring**			
Data monitoring	21a	Composition of data monitoring committee (DMC); summary of its role and reporting structure; statement of whether it is independent from the sponsor and competing interests; and reference to where further details about its charter can be found, if not in the protocol. Alternatively, an explanation of why a DMC is not needed	Data Management & Ethics and Dissemination & Declarations
	21b	Description of any interim analyses and stopping guidelines, including who will have access to these interim results and make the final decision to terminate the trial	Data Management & Ethics and Dissemination
Harms	22	Plans for collecting, assessing, reporting, and managing solicited and spontaneously reported adverse events and other unintended effects of trial interventions or trial conduct	Data Management

Auditing	23	Frequency and procedures for auditing trial conduct, if any, and whether the process will be independent from investigators and the sponsor	Quality Assessment
**Ethics and dissemination**			
Research ethics approval	24	Plans for seeking research ethics committee/institutional review board (REC/IRB) approval	Declarations
Protocol amendments	25	Plans for communicating important protocol modifications (eg, changes to eligibility criteria, outcomes, analyses) to relevant parties (eg, investigators, REC/IRBs, trial participants, trial registries, journals, regulators)	Ethics and Dissemination
Consent or assent	26a	Who will obtain informed consent or assent from potential trial participants or authorised surrogates, and how (see Item 32)	Methods, Recruitment
	26b	Additional consent provisions for collection and use of participant data and biological specimens in ancillary studies, if applicable	Not applicable
Confidentiality	27	How personal information about potential and enrolled participants will be collected, shared, and maintained in order to protect confidentiality before, during, and after the trial	Data Management
Declaration of interests	28	Financial and other competing interests for principal investigators for the overall trial and each study site	Declarations
Access to data	29	Statement of who will have access to the final trial dataset, and disclosure of contractual agreements that limit such access for investigators	Data Management
Ancillary and post-trial care	30	Provisions, if any, for ancillary and post-trial care, and for compensation to those who suffer harm from trial participation	Not applicable
Dissemination policy	31a	Plans for investigators and sponsor to communicate trial results to participants, healthcare professionals, the public, and other relevant groups (eg, via publication, reporting in results databases, or other data sharing arrangements), including any publication restrictions	Ethics and Dissemination

For peer review only

	31b	Authorship eligibility guidelines and any intended use of professional writers	Not applicable
	31c	Plans, if any, for granting public access to the full protocol, participant-level dataset, and statistical code	Ethics and Dissemination
**Appendices**			
Informed consent materials	32	Model consent form and other related documentation given to participants and authorised surrogates	Available on request
Biological specimens	33	Plans for collection, laboratory evaluation, and storage of biological specimens for genetic or molecular analysis in the current trial and for future use in ancillary studies, if applicable	Not applicable.

*It is strongly recommended that this checklist be read in conjunction with the SPIRIT 2013 Explanation & Elaboration for important clarification on the items. Amendments to the protocol should be tracked and dated. The SPIRIT checklist is copyrighted by the SPIRIT Group under the Creative Commons "Attribution-NonCommercial-NoDerivs 3.0 Unported" license.

For peer review only
